# Long-Term Evaluation and Normal Tissue Complication Probability (NTCP) Models for Predicting Radiation-Induced Optic Neuropathy after Intensity-Modulated Radiation Therapy (IMRT) for Nasopharyngeal Carcinoma: A Large Retrospective Study in China

**DOI:** 10.1155/2022/3647462

**Published:** 2022-02-23

**Authors:** Yan-Ling Wu, Wen-Fei Li, Kai-Bin Yang, Lei Chen, Jing-Rong Shi, Fo-Ping Chen, Xiao-Dan Huang, Li Lin, Xiao-Min Zhang, Jing Li, Yu-Pei Chen, Ling-Long Tang, Yan-Ping Mao, Jun Ma

**Affiliations:** ^1^Department of Radiation Oncology, Sun Yat-sen University Cancer Center, State Key Laboratory of Oncology, South China, Collaborative Innovation Center for Cancer Medicine, Guangdong Key Laboratory of Nasopharyngeal Carcinoma Diagnosis and Therapy, Guangzhou 510060, China; ^2^Department of Data Mining and Analysis, Guangzhou Tianpeng Technology Co. Ltd., Zhujiang East Rd. No. 11, Guangzhou 510627, China

## Abstract

**Purpose:**

To quantify the long-term evaluation of optic chiasma (OC) and/or optic nerve(s) (ONs) and to develop predictive models for radiation-induced optic neuropathy (RION) in nasopharyngeal carcinoma after intensity-modulated radiotherapy (IMRT).

**Methods and Materials:**

A total of 3,662 patients' OC/ONs with full visual acuity and dosimetry data between 2010 and 2015 were identified. Critical dosimetry predictors of RION were chosen by machine learning and penalized regression for survival. A nomogram containing dosimetry and clinical variables was generated for predicting RION-free survival.

**Results:**

The median follow-up was 71.79 (2.63–120.9) months. Sixty-six eyes in 51 patients (1.39%) developed RION. Two patients were visual field deficient, and 49 patients had visual acuity of less than 0.1 (20/200). The median latency time was 36 (3–90) months. The 3-, 5-, and 8-year cumulative incidence of RION was 0.78%, 1.19%, and 1.97%, respectively. Dmax was the most critical dosimetry variable for RION (AUC: 0.9434, the optimal cutoff: 64.48 Gy). Patients with a Dmax ≥64.48 Gy had a significantly higher risk of RION (HR = 102.25; 95%CI, 24.86–420.59; *P* < 0.001). Age (>44 years) (HR = 2.234, 95% CI = 1.233–4.051, *p* = 0.008), advanced *T* stage (T3 vs. T1-2: HR = 7.516, 95% CI = 1.725–32.767, *p*=0.007; T4 vs. T1-2: HR = 37.189, 95% CI = 8.796–157.266, *P* < 0.001), and tumor infiltration/compression of the OC/ONs (HR = 4.572, 95% CI = 1.316–15.874, *p*=0.017) were significant clinical risk factors of RION. A nomogram comprising age, *T* stage, tumor infiltration/compression of the OC/ON, and Dmax significantly outperformed the model, with only Dmax predicting RION (C-index: 0.916 vs. 0.880, *P* < 0.001 in the training set; 0.899 vs. 0.874, *P*=0.038 in the test set). The nomogram-defined high-risk group had a worse 8-year RION-free survival.

**Conclusions:**

In the IMRT era, *D*max <60 Gy is safe and represents an acceptable dose constraint for most NPC patients receiving IMRT. A reasonable trade-off for selected patients with unsatisfactory tumor coverage due to proximity to the optic apparatus would be Dmax <65 Gy. Caution should be exercised when treating elderly and advanced *T*-stage patients or those with tumor infiltration/compression of the OC/ON. Our nomogram shows strong efficacy in predicting RION.

## 1. Introduction

Radiation-induced optic neuropathy (RION) is a rare and catastrophic late-onset complication after radiation therapy (RT) to the optic chiasma (OC) and/or optic nerve(s) (ONs) in head and neck cancer (HNC). It usually presents as acute, painless, progressive, and irreversible loss of vision or hemianopsia in one or both eyes in the months to years after treatment [1]. In 1972, L J Shukovsky detailed eye complications in 15 HNC patients treated with RT, and three patients lost sight due to RION [2]. Due to the severity and irreversibility of RION, the Radiation Therapy Oncology Group (RTOG) 0615 trial recommended a maximum point dose (*D*max) of ≤50 Gy (biologically equivalent dose (EQD_2Gy_)) for the whole OC or ONs [3]. Unlike 2D-RT, intensity-modulated radiotherapy (IMRT) enables highly accurate positioning and precise dosimetry information, significantly reducing RT-related side effects [4]. In a recent study of 125 patients with a Dmax to the anterior visual pathway of ≥50 Gy undergoing IMRT, five patients were diagnosed with RION. In all five patients, visual acuity post-IMRT had only mildly decreased [5]. Therefore, it appears that the incidence of RION remains low even if patients exceed the recommended dose constraint. Thus, the question of whether the recommended dose constraint for the OC/ONs is relatively conservative in the modern IMRT era possibly diminishes the probability of a cancer cure.

Curative treatments do not exist for RION, so a greater understanding of the etiological factors triggering RION is required [6]. There is consensus on the radiation dose as the most important risk factor. However, clinical risk factors have remained elusive and hard to verify [5,7–13]. A more in-depth dosimetry analysis and a risk factor of nomogram are still lacking. Yet, there is unanimous agreement that the OC and the unilateral ON should be included as a priority 1 for dose constraint [14]. If bilateral blindness from damage to the OC or both ONs is unavoidable to achieve an adequate dose to cover the target tumor area, the most suitable approach should be discussed with the patient. A preferable dose constraint is crucially important to optimize tumor control while balancing the risk of RION. Meanwhile, a reliable nomogram will help clinicians and patients to make more informed decisions.

While nasopharyngeal carcinoma (NPC) is globally rare, it has a high incidence in southern China [15]. In contrast to other HNCs, it is anatomically adjacent to the OC and ONs and RT is the primary curative treatment with a 5-year overall survival rate of 82% without distant metastasis, meaning NPC is a suitable candidate for studying RION [16]. For advanced NPC invading the base of the skull and extending into the cavernous sinus and paraorbital regions, the OC and ONs are usually included in the radiation field and are routinely treated with a radiation dose of 66 Gy or higher (EQD_2Gy_ is often greater than 70 Gy). Beyond the tolerated dose range, RION is the most serious complication in these patients. Here, we investigate the largest NPC clinical database on RION treated with IMRT with a long-term follow-up to (1) quantify the tolerance of the OC and ONs, (2) identify the long-term risk factors for a new dose constraint, and (3) generate a nomogram for predicting RION.

## 2. Materials and Methods

### 2.1. Patient Selection

All NPC patients treated with IMRT diagnosed between October 2009 and December 2015 were searched from the previously introduced NPC-specific big data intelligence platform at the Sun Yat-sen University *Cancer* Center (SYSUCC) [17]. A total of 3,662 patients with full visual acuity and detailed dosimetry data were selected. A flowchart of the patient inclusion criteria is shown in Supplementary Figure 1. The authenticity of this study has been validated by uploading the key raw data onto the Research Data Deposit public platform (http://www.researchdata.org.cn) (RDDA2022229679). This project was approved by SYSUCC's institutional review committee (B2020-142-01).

### 2.2. Treatment

All patients received radical high-total and fractionated-dose simultaneous modulated accelerated radiotherapy boost IMRT. Radiation planning was performed utilizing high-resolution, contrast-enhanced CT under image-guided conditions with reliable contouring and a well-documented 3D dosimetry plan and a scheduled total dose of 68–74 Gy (EQD_2Gy_ = 73.2–81.1 Gy) in 30–34 fractions [18]. The treatment for stage I disease was RT alone. For stage II-IVB disease, it was RT plus concurrent chemotherapy (CC), with or without induction chemotherapy (IC) or adjuvant chemotherapy (AC) [19]. The normal tissue constraints were according to RTOG 0225 and 0615 and the quantitative analysis of normal tissue effects in the clinic (QUANTEC) [3, 20].

### 2.3. Evaluation of Dosimetry and Vision

Summation plans and dose-volume histograms (DVHs) were available for every patient. There was no planning organ at risk volume (PRV) around the OC or ONs. To enable comparisons within this study and the literature, we calculated the biologically equivalent dose (EQD_2Gy_) with an *α*/*β* value of 10 Gy for the tumor and 1.6 Gy for the OC and ONs [21].

Follow-up duration was calculated from the day visual acuity data were collected. Patients were followed up every three months during the first three years and then every six months thereafter. Visual acuity-deficient patients during the follow-up were referred to an ophthalmologist. Patients and their family members were contacted to obtain whether they had eye diseases before and after IMRT. Patients with eye diseases were asked to provide ophthalmology visit records or optometry results.

Best-corrected visual acuity (BCVA) was the main outcome and measured using the standard Snellen chart in China. BCVA was split into five grades: 0.8 or better (≥20/25); 0.4 to 0.7 (20/50 to 20/30); 0.1 to 0.3 (20/200 to 20/60); and less than 0.1 (20/200) to greater than counting fingers and counting fingers to no light perception [22]. The 5-point BCVA scale based on the information provided by the patients and family members was completed and checked by two senior radiotherapists.

### 2.4. Definition of RION

RION as an endpoint was defined as visual field deficiency or deterioration of the BCVA by one grade from the time of IMRT, regardless of initial visual status. Severe RION was defined as visual field deficiency or blindness of an eye or deterioration of the BCVA by two grades. The exclusion criteria for RION diagnosis were as follows: 1. tumor-related visual acuity deficient, such as recurrence of NPC; 2. other eye diseases that may cause loss of vision, such as multiple sclerosis, cataracts, glaucoma, or vasculopathy due to diabetes or hypertension. The latent time to RION was measured in months from the beginning of IMRT to the commencement of any visual acuity deficiency.

### 2.5. Statistical Analysis

Statistical analyses were performed using SPSS v22.0 (IBM Corp, Armonk, NY) or “R” version 4.0.4 (https://www.r-project.org/). Curves for side effects were calculated using the Kaplan-Meier analyses and compared using the log-rank tests. Group comparisons were conducted using the chi-square test or Fisher's exact test for categorical variables, and the Student's *t*-test for continuous variables.

The recursive feature elimination (RFE), boosting in Cox regression (CoxBoost), random forests, regression and classification (RF-SRC), and least absolute shrinkage and selection operator (LASSO) logistic regression for survival analysis identified critical predictive dosimetry parameters for RION. A detailed description of these methods and the *R* codes used were recorded in the Supplementary Materials. The area under the receiver operating curve (AUC) for 3-, 5-, and 8-year was calculated. Univariate and multivariate Cox proportional hazards models were used to select clinical risk factors. Hazard ratios (HRs) and 95% confidence intervals (CIs) were calculated in the Cox regression analysis. A normal tissue complication probability (NTCP) model was constructed with the selected clinical risk factors and the most predictive dosimetry parameter. The performance of the NTCP model was compared with only the most predictive dosimetry parameter with Harrell's concordance index (C-index). A two-sided *P* value < 0.05 was considered statistically significant in all the above analyses.

## 3. Results

### 3.1. The Evolution of RION

The median follow-up was 71.79 (2.63–120.9) months. A detailed description of the evolution of visual acuity was recorded in the Supplementary Materials. The BCVA grades 1–5 for visual acuity-deficient disease distribution pre-IMRT and post-IMRT are shown in Supplementary Figure 2. Post-IMRT, the BCVA deterioration grades ≥1 compared with baseline were 72 eyes in 55 (1.5%) patients and visual field deficiency in three eyes in two (0.1%) patients. None of the excluded patients (*n* = 6, eyes = 7) had deterioration associated with radiotherapy. Reasons for deterioration in six patients with other events were local recurrence, diabetic retinopathy, central retinal vein occlusion, maculopathy, viral optic neuritis, and fundus hemorrhage, respectively. The patient with maculopathy also had cataracts, thus two factors contributing to their visual acuity deterioration. Eventually, 68 eyes in 51 (1.39%) patients were diagnosed with RION.

Fifty-one patients had all severe RION. Hemianopsia patients (*n* = 2, both eyes = 1) presented symptoms with temporal field loss (left side) and generalized constricted fields (both eyes), respectively. The BCVA deteriorated ≥2 grade patients (*n* = 49, both eyes = 16) all presented with a visual acuity less than 0.1 (20/200) in which 24 were blind (both eyes = 4). The median latency time was 36 (3–90) months. One patient was thought to suffer from loss of vision (left side) due to tumor optic nerve compression and edema of the surrounding tissue during IMRT earlier than would be expected for the development of RION. The patient improved with steroids but was blind three months after the completion of IMRT. Details on patients with decreased visual acuity associated with RION are shown in Supplementary Table 1. Supplementary Figure 3 shows the tumor, IMRT dosimetry plan, and MRI of RION blindness (both eyes) in one of the patients.

**Figure 1:**
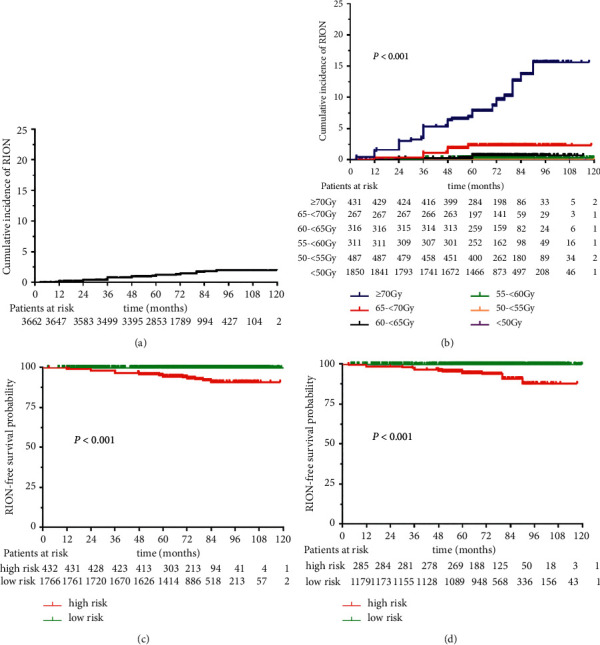
Actual cumulative risk of RION over time (a) stratified by radiation dosage (b). RION-free survival probability curves of the low-risk and high-risk groups stratified by the nomogram in the training (c) and test sets (d). RION = radiation-induced optic neuropathy.

In the entire cohort, the 3-year, 5-year, and 8-year cumulative incidence of RION was 0.78%, 1.19%, and 1.97%, respectively. In the Dmax dose ≥70 Gy, 65-<70 Gy, 60-<65 Gy, 55-<60 Gy, 50-<55 Gy, and <50 Gy groups, the 3-year cumulative incidence of RION was 5.34%, 1.12%, 0.32%, 0.33%, 0%, and 0%, respectively; the 5-year cumulative incidence was 7.83%, 2.36%,0.7%, 0.33%, 0%, and 0%, respectively; and the 8-year cumulative incidence was 15.59%, 2.36%, 0.7%, 0.33%, 0%, and 0%, respectively. The incidence of RION curves over time and stratified by dose are shown in Figure 1(a) and 1(b). Details on dose-level stratifications are shown in Supplementary Table 2.

### 3.2. Patient Characteristics

The median age was 44 (7–81) years old, and 2,730 (74.5%) patients were men. Tumors were adjacent to the OC in 2,831 (77.3%) patients and the ONs in 831 (22.7%) patients. Common risk factors included DM (4.5%), hypertension (5.2%), alcohol consumption (17.2%), and smoking (33.5%). The treatment was a combination of radiotherapy and chemotherapy. RT alone, IC + CC group (IC ± CC ± AC ± target), and CC group (CC ± AC ± target) were 488 (14.5%), 1764 (48.7%), and 1,410 (38.9%) patients, respectively. Tumor-related visual acuity deficiency before therapy was present in 106 (2.9%) patients including 19 (0.5%) patients with tumor infiltration/compression of the OC/ON. Note that some visual acuity-deficient patients before therapy were related to tumors but unaffected for the BCVA in one eye (ocular motility disorder = 9, diplopia = 48, ptosis = 6, other = 4, and multiple symptoms = 16); only tumor infiltration/compression of the OC/ON will cause deterioration of the BCVA grade in one eye in our study. In total, 51 RION patients and the remaining 3,611 patients without RION were used as a comparison group for the dosimetry analysis. For baseline RION and non-RION patient characteristics, see Table 1.

### 3.3. Dosimetry Factor Analyses

**Figure 2:**
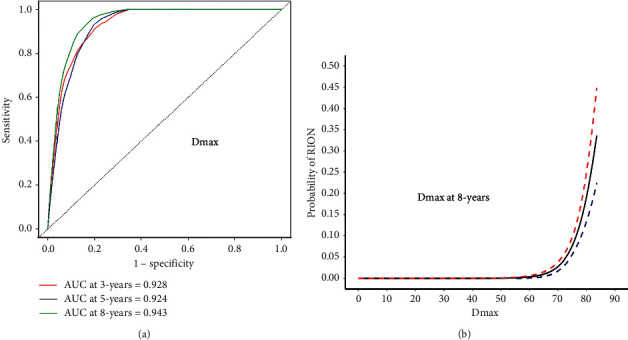
The time-dependent receiver operating characteristic (ROC) curve (a) and dose-effect curves (b) of Dmax for RION. Dmax had the highest AUC value at 8 years with 0.9434. The optimal cutoff of Dmax was 64.48 Gy (sensitivity = 0.955; specificity = 0.814). Solid and dashed lines indicate logistic regression and the 95% CI dose tolerance, respectively. RION = radiation-induced optic neuropathy; *D*max = maximum point dose; AUC = area under the receiver operating characteristic curve; CI = confidence intervals.

A comparison of dosimetry parameters between RION and non-RION patients is presented in Supplementary Table 3. The Spearman correlation matrix in Supplementary Figure 4 shows strong correlations between 20 dosimetry parameters. The RFE, CoxBoost, RF-SRC, and LASSO were used to choose critical dosimetry factors (Table 2 and Supplementary Figures 5–7). The 3-, 5-, and 8-year area under the receiver operating curve (AUC) and optimal corresponding sensitivity and specificity cutoff were calculated (Supplementary Table 5). The Dmax with the highest AUC value at 8 years was 0.9434 with the optimal cutoff 64.48 Gy (sensitivity = 0.955; specificity = 0.814) (the time-dependent receiver operating characteristic curve is shown in Figure 2(a); dose-effect curve is shown in Figure 2(b); and time-dependent AUC is shown in Supplementary Figure 8). Patients with a Dmax ≥64.48 Gy at 8 years had a significantly higher risk of RION than those with a Dmax <64.48 Gy (hazard ratio (HR) = 102.25; 95% confidence interval (CI), 24.86–420.59; *P* < 0.001).

### 3.4. Clinical Factor Analyses

We analyzed clinical characteristics that might be associated with RION. There were low-to-moderate correlations between Dmax and clinical factors, with the highest Spearman coefficient of 0.53 (Supplementary Figure 9). Age (>44 years) (HR = 2.234, 95% CI = 1.233–4.051, *p* = 0.008), advanced *T* stage (T3 vs. T1-2: HR = 7.516, 95% CI = 1.725–32.767, *p* = 0.007; T4 vs. T1-2: HR = 37.189, 95% CI = 8.796–157.266, *p* < 0.001), and tumor infiltration/compression of the OC/ONs (HR = 4.572, 95% CI = 1.316–15.874, *p* = 0.017) were associated with an increased risk of RION (Supplementary Figure 10).

### 3.5. NTCP Models for Predicting RION

**Figure 3:**
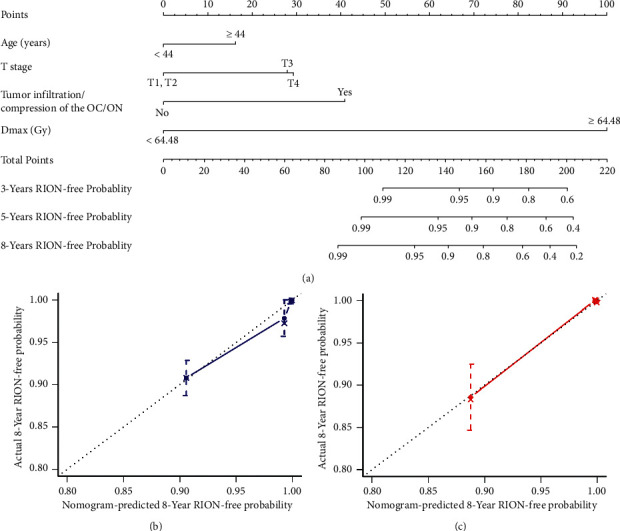
A nomogram for predicting 3-, 5-, and 8-year RION-free survival (a); calibration curves of the nomogram (b) in the training and (c) the test sets. RION = radiation-induced optic neuropathy; *D*max = maximum point dose.

The RION dataset was randomly divided into a training set (60%) and a test set (40%) for the explorative construction and validation of NTCP models for predicting RION. A nomogram was constructed with the selected clinical factors (age, *T* stage, and tumor infiltration/compression of the OC/ONs) and the most critical dosimetry variables (*D*max) in the training set (Figure 3(a)). There were low-to-moderate correlations between these significant risk variables, with the highest Spearman coefficient of 0.53 (Supplementary Figure 11). Detailed point assignment of the nomogram is described in Supplementary Table 6. The C-index of the nomogram was significantly better than the model with Dmax only in both the training set (0.916 vs. 0.880, *P* < 0.001) and the test set (0.899 vs. 0.874, *P*=0.038). The calibration curves for 8-year RION-free probability were close to the 45-degree lines in both the training set and the test set (Figure 3(b) and 3(c)), indicating that the occurrence of RION could be accurately predicted with our nomogram. Using 116 as the cutoff value of the nomogram-generated scores, the patients in the training set and the test set were divided into high-risk and low-risk groups. Eight-year RION-free survival was significantly worse in the high-risk group compared with the low-risk group in the training set (90.8% vs. 99.9%; HR = 60.884, 95% CI: 14.523–255.244, *P* < 0.001; Figure 1(c)) and the test set (87.8% vs. 99.9%; HR = 81.127, 95% CI: 10.845–606.858, *P* < 0.001; Figure 1(d)).

## 4. Discussion

RION has not been well defined in the IMRT era. In this study, Dmax was the most critical dosimetry variable for RION, and a dose of <64.48 Gy was the optimal cut point for the Dmax in our dataset. Until further prospective datasets become available, our cut point may represent an acceptable upper limit for the Dmax in HNC patients treated with IMRT. To our knowledge, this is the first and largest study to analyze the relationship between RION and dosimetry parameters for developing NTCP models with dosimetry and clinical risk factors for RION after IMRT.

### 4.1. The Evolution of RION

At present, the detailed pathogenesis responsible for RION remains unclear. Many believe it is a consequence of direct injury to nervous tissue. Some feel that its ischemic origin is due to vascular injury, and for others, there is conjecture about an autoimmune mechanism [3, 7, 23]. The first signs of RION are fundoscopically characterized by swelling of the optic disc, flame-shaped peripapillary hemorrhages, hard exudates, and subretinal fluid [24]. T1-weighted enhanced magnetic resonance imaging (MRI) usually shows contrast-enhanced lesions of the affected OC or ONs [25]. However, patient symptoms often present weeks later. Ultimately, RION is characterized by an atrophied pale optic disc and atrophied optic nerve with irreversible vision loss. Visual field defects consist of a nerve fiber bundle or chiasmal defects [1].

In our study, the evolution of RION generally conforms to previously reported cases [1]. In the course of their visual deterioration, none of the patients had swelling of the eye or reported eye pain. Visual loss was irreversible and tended to aggravate over a longer follow-up. Previous studies have shown that RION can occur anywhere from three months to nine years or longer, but the majority present within three years, with a peak incidence at one to one and a half years [26,27]. Similarly, the onset of RION appeared at a median of 36 months (3–90 months) after IMRT in our study.

In traditional RT studies, when the ONs or OC are exposed to radiation, an estimated 75% of patients will have vision loss in both eyes [28]. However, 20 (39.2%) out of 51 RION patients had bilateral vision loss in our study. Four RION patients that are blind in both eyes (7.8%) occurred in the Dmax ≥70 Gy group only (Supplementary Table 2). Our data also show a lower occurrence of RION than reported by the QUANTEC initiative, reporting expected risks of <3%, 3–7%, and 7–20% of RION in the dose ranges of Dmax <55 Gy, 55–60 Gy, and >60 Gy, respectively [7]. Susan Brecht et al. [5] show that in 125 patients with a Dmax to the anterior visual pathway of ≥50 Gy (average: 53.1 ± 3.9 Gy) undergoing IMRT, five patients were diagnosed with mild RION. Four, zero, and one of RION occurred in the dose ranges of Dmax <55 Gy, 55–60 Gy, and >60 Gy, respectively. Puyao C. Li [10] shows that 514 patients were treated with proton and photon therapy, who received a minimum of 30 Gy (relative biologic effectiveness (RBE)) to the anterior optic pathway. A total of 17 patients (3.3%) developed RION. Cumulative incidence of RION was 1% among patients receiving <60 Gy (RBE) and 5.8% among patients receiving ≥60 Gy (RBE) to the optic pathway, indicating that the incidence and severity of RION might be mitigated by modern IMRT.

### 4.2. Dosimetry and Clinical Risk Factor Considerations

Existing recommendations for the OC and ONs are relatively conservative based on conventional RT data [3,7]. New dose criteria in the IMRT era need to be established. The development of RION mostly depends on DVH-associated factors (Supplementary Table 4). However, owing to the general multicollinearity problem among these factors, it is hard to identify a singularly critical dosimetry predictor (Supplementary Figure 4). We applied the RFE, CoxBoost, RF-SRC, and LASSO methods in our study and identified Dmax as the most critical dosimetry predictor. The time-dependent AUC shows that the AUC of Dmax continues to increase after 5 years (Supplementary Figure 8). Thus, a longer observation time may be necessary to reveal true RION rates. Dmax to OC and ONs should always follow the ALARP (as low as reasonably practicable) principles. In our study, *D*max <60 Gy is safe (Figure 2(b)) and represents an acceptable dose constraint for most NPC patients receiving IMRT [14]. *D*max had the highest AUC value at 8 years with 0.9434 and an optimal cutoff of 64.48 Gy. Therefore, a reasonable trade-off for selected patients with unsatisfactory tumor coverage due to proximity to the optic apparatus would be Dmax <65 Gy. The risks of RION for Dmax <65 Gy, the 3-year, 5-year, and 8-year were 0.07%, 0.11%, and 0.11%, respectively.

Considering the large weight of dosimetry factors, and a moderate linear relationship between the most important dosimetry factor (*D*max) and the clinical risk factor (T stage) (Supplementary Figure 9), it will affect the results of univariate and multivariate analysis for clinical factors when Dmax was incorporated. In order to better screen out the critical dosimetry and clinical risk factors, we divided them into two parts and combined them into the NTCP model. When tumors are large and compressed or directly infiltrate the OC or ONs, high-dose irradiation is inevitable. In our study, the 8-year incidence of RION was higher in patients with T3 (1.50%) and T4 (8.36%) disease than in those with T1-T2 disease (0.14%). The small number of RION events limited the statistical power of this study in analyzing other potential risk factors. For example, diabetes mellitus and hypertension were not found to be risk factors, though this may merely reflect their low prevalence in our cohort.

In our study, additional clinical risk factors improved the predictive ability of the DVH-based model for RION, as shown by the larger C-index. The nomogram, which included age, *T* stage, tumor compression/infiltration of the OC/ON, and Dmax, showed good discrimination and calibration (Figure 3). However, more studies are needed to validate its efficacy. Identifying risk factors for developing RION and NTCP models could inform planning, monitoring, and follow-up for high-risk patients.

### 4.3. Limitations

The limitations of this study mainly stem from its retrospective nature. First, 528 patients failed to provide full visual acuity data. Lost to follow-up or death patients might have been at an advanced stage, elderly, and/or having severe side effects. Such missing data may underestimate the risk of RION. Second, because there is no universal, regular, and comprehensive eye examination for patients, the diagnosis of subclinical RION events is difficult to assess. However, other published RION studies have such limitations. At the same time, subclinical RION events were not the focus of this study as they have a lesser impact on the patient's quality of life. Third, this was a single institutional study. Large multicenter prospective studies are necessary to validate our findings.

## 5. Conclusions

The prevalence of RION remains low after IMRT. Dmax to OC and ONs should always follow the ALARP principles. *D*max <60 Gy is safe and represents an acceptable dose constraint for most NPC patients receiving IMRT. A reasonable trade-off for selected patients with unsatisfactory tumor coverage due to proximity to the optic apparatus would be Dmax <65 Gy. Yet, caution should be exercised when treating elderly and advanced *T*-stage patients or those with tumor infiltration/compression of the OC/ONs. Our nomogram may accurately predict RION and allow for the follow-up management of patients. Further prospective multicenter studies are needed to validate or complement our findings.

## Figures and Tables

**Table 1:** Baseline characteristics.

Baseline patient factor or risk factor		RION (%) n = 51	Non-RION (%) n = 3,611	☼ P value
Age (years) Median (min∼max)		50 (16∼73)	44 (7∼81)	0.120
Gender	Female	18 (35.3)	914 (25.3)	0.104
	Male	33 (64.7)	2,697 (74.7)	
Diabetes mellitus	Yes	3 (5.9)	164 (4.5)	0.649
	No	48 (94.1)	3,447 (95.5)	
Hypertension	Yes	5 (9.8)	186 (5.2)	0.138
	No	46 (90.2)	3,425 (94.8)	
Alcohol consumption	Yes	10 (19.6)	628 (17.4)	0.679
	No	41 (80.4)	2,983 (82.6)	
Smoking	Yes	18 (35.3)	1,209(33.5)	0.785
	No	33 (64.7)	2,402 (66.5)	
Tumor localization	Chiasm/midline	38 (74.5)	2,793 (77.3)	0.839
	Left optic nerve	7 (13.7)	403 (11.2)	
	Right optic nerve	6 (11.8)	415 (11.5)	
T stage (AJCC, 8th ed.)	T1-2	2 (3.9)	1,513 (41.9)	<0.001
	T3	16 (31.4)	1,564 (43.3)	
	T4	33( 64.7)	534 (14.8)	
Stage (AJCC, 8th ed.)	I-II	2 (3.9)	1,054 (29.2)	<0.001
	III	13 (25.5)	1,648 (45.6)	
	IVA	36 (70.6)	909 (25.2)	
EBV DNA before treatment	≥2,000	30 (58.9)	1,578 (44.3)	
(Copy/ml)	<2,000	21 (41.2)	1,983 (55.7)	0.019
☆Treatment	RT alone	4 (7.8)	484 (13.4)	0.005
	IC + CC group	37 (72.5)	1,727 (47.8)	
	CC group	10 (19.6)	1,400 (38.8)	
Best-corrected visual acuity deficiency at the start of therapy		5 (9.8)	20 (0.6)	<0.001
*∗* Tumor infiltration/compression of the chiasm/optic nerves		5 (9.8)	14 (0.4)	<0.001
♯Any other factors affecting best-corrected visual acuity eye diseases		0	6 (0.2)	<0.001

☆The treatment was a combination of radiotherapy and chemotherapy, RT = radiation therapy, IC = induction chemotherapy, CC = concurrent chemotherapy, AC= adjuvant chemotherapy; IC + CC group (IC + CC=1,229, IC + CC + AC= 61, IC + CC + target = 157, IC + CC + AC + target = 3, IC = 285, IC + AC = 16, IC + target = 13); CC group (CC= 1,250, CC + AC = 71, CC + target = 89). *∗*Note that some visual acuity-deficient patients at the start of therapy were related to tumors but unaffected for the best-corrected visual acuity in one eye (ocular motility disorder = 9, diplopia = 48, ptosis = 6, other = 4, and multiple symptoms = 16); only tumor infiltration/compression of the optic nerves/chiasm will cause deterioration of the BCVA grade in one eye in our study. ♯Any other affects the BCVA eye diseases at the start of therapy (ocular trauma = 1, retinal disease = 4, and cataracts = 1). ☼P-values were calculated using the chi-square test or independent t-test if indicated. Abbreviations: EBV = Epstein-Barr virus; BCVA = best-corrected visual acuity.

**Table 2:** Important dosimetric features and variables selected by the machine learning and LASSO.

	RFE	CoxBoost	RF-SRC	LASSO
1	Dmax	Dmax	Dmax	D50
2	D50	D50	D35	Dmin
3	/	D35	D1	/

Abbreviations: RFE = recursive feature elimination; CoxBoost = boosting in Cox regression; RF-SRC = random forests for survival, regression, and classification; LASSO = least absolute shrinkage and selection operator; Dmin = minimum point dose; Dmax = maximum point dose; D1-50 = minimum dose 1%-50% volume of the optic nerve or optic chiasma.

## Data Availability

Research data are stored in the Research Data Deposit public platform (http://www.researchdata.org.cn) (RDDA2022229679).

## Supplementary Materials

Supplementary Materials The supplementary document includes a summary, supplementary methods and results, supplementary figures, supplementary tables, and supplementary *R* codes. Among them, supplementary methods and results include patients restaging, evaluations before diagnosis and treatment, models for selecting significant dosimetry predictors (RFE, CoxBoost, RF-SRC, and LASSO), and the evolution of visual acuity and only show the *R* codes of the RFE, CoxBoost, RF-SRC, and LASSO for selection of important dosimetric variables. Supplementary Figure 1. Flowchart of patient selection. Abbreviations: NPC = nasopharyngeal carcinoma; IMRT = intensity-modulated radiotherapy; *D*max = maximum point dose; RT = radiation therapy; LASSO = the least absolute shrinkage and selection operator; AUC = area under the receiver operating characteristic curve. Supplementary Figure 2. Pre-IMRT and post-IMRT for best-corrected visual acuity disease distribution are shown for the left and right eyes on a 5-grade scale. Abbreviations: IMRT = intensity-modulated radiation therapy. Supplementary Figure 3. An example of RION blindness (both eyes). (A) An advanced NPC invading the paraorbital regions (green arrow) and a patient with diplopia at the start of therapy; (B) IC + CCRT treatment: IC was TPF regimen for 3 cycles, CC was cisplatin for 2 cycles, and a scheduled total dose of 70.08 Gy (EQD_2Gy_ = 73.78 Gy) in 32 fractions with the OC and ONs was included in the radiation field with a Dmax of the OC of 78.52 Gy (EQD_2Gy_ = 82.66 Gy); and (C) 24 months after IMRT, both eyes presented acute, painless, progressive, and irreversible loss of vision within four months, and the OC was enhanced in T1WI + *C* (red arrow). TPF (cisplatin (60 mg/m^2^) with 5-fluorouracil (600 mg/m^2^ over 120 h) and docetaxel (60 mg/m^2^)); cisplatin (100 mg/m^2^) given in weeks 1 and 4 of radiotherapy. Abbreviations: RION = radiation-induced optic neuropathy; RT = radiation therapy, IC = induction chemotherapy, CC = concurrent chemotherapy; *D*max = maximum point dose; OC = optic chiasma; ON = optic nerve; T1WI + *C* = postcontrast T1-weighted image. Supplementary Figure 4. Spearman's correlation matrix of 20 dosimetry factors. Value means Spearman's correlation coefficient. It shows a strong correlation with dosimetry factors. Dmin = minimum point dose; Dmean = mean dose; *D*max = maximum point dose; D1-50 = minimum dose 1%–50% volume of the optic nerve or optic chiasma; D0.01–0.09 cc = minimum dose to 0.01–0.09 cc volume of the optic nerve or optic chiasma. Supplementary Figure 5. Coefficient paths for the 20 dosimetry factors using boosting in Cox regression. Optional covariates with nonzero coefficients at boosting Step 500: parameter estimate >0: Dmin, *D*max, D25, D35, D50, parameter estimate <0: Dmean, D0.07 cc, D0.09 cc. Dmin = minimum point dose; Dmean = mean dose; *D*max = maximum point dose; D1-50 = minimum dose 1%–50% volume of the optic nerve or optic chiasma; D0.01–0.09 cc = minimum dose to 0.01–0.09 cc volume of the optic nerve or optic chiasma. Supplementary Figure 6. Important variables were selected from the 20 dosimetry factors using the random survival forest model. Dmin = minimum point dose; Dmean = mean dose; *D*max = maximum point dose; D1-50 = minimum dose 1%–50% volume of the optic nerve or optic chiasma; D0.01–0.09 cc = minimum dose to 0.01–0.09 cc volume of the optic nerve or optic chiasma. Supplementary Figure 7. Plot of LASSO coefficients and selective variables at lambda.1se (0.0432). LASSO = least absolute shrinkage and selection operator. Dmin = minimum point dose; Dmean = mean dose; *D*max = maximum point dose; D1-50 = minimum dose 1%–50% volume of the optic nerve or optic chiasma; D0.01–0.09 cc = minimum dose to 0.01–0.09 cc volume of the optic nerve or optic chiasma. Supplementary Figure 8. The lines depict the time-dependent AUC for *D*max. Abbreviations: AUC = area under the receiver operating characteristic curve. *D*max = maximum point dose. Supplementary Figure 9. Spearman's correlation matrix between significant clinical variables and the most important dosimetry predictor: Dmax. Value means Spearman's correlation coefficient. There were low-to-moderate correlations between Dmax and the significant clinical variables, with the highest Spearman coefficient: 0.53. Abbreviations: Dmax = maximum point dose; OC = optic chiasma; ON = optic nerve; EBV = Epstein-Barr virus. Supplementary Figure 10. Forest plots depicting the univariate and multivariate associations of clinical factors with RION. Squares represent aHRs with 95% CIs indicated by the horizontal bars. The treatment was a combination of radiotherapy and chemotherapy, IC + CC group = IC ± CC ± AC ± target; CC group = CC ± AC ± target. Abbreviations: RION = radiation-induced optic neuropathy; aHR = adjusted hazard ratio; CIs = confidence intervals; EBV = Epstein-Barr virus; RT = radiation therapy, IC = induction chemotherapy, CC = concurrent chemotherapy, AC = adjuvant chemotherapy. Supplementary Figure 11. Spearman's correlation matrix between significant clinical variables and *D*max. Value means Spearman's correlation coefficient. There were low-to-moderate correlations between Dmax and the significant clinical variables, with the highest Spearman coefficient: 0.53. Abbreviations: Dmax = maximum point dose; OC = optic chiasma; ON = optic nerve. Supplementary Table 1: details on patients with decreased visual acuity associated with RION. Supplementary Table 2: dose-level stratifications for Dmax to the OC/ON. Supplementary Table 3: comparison of dosimetry parameters between RION and non-RION patients. Supplementary Table 4: univariate analysis for the dosimetry parameters predicting the development of RION. Supplementary Table 5: the time-dependent AUC and cutoff values of the selected variables. Supplementary Table 6: point assignment of the nomogram for RION in the overall analysis.
