# Supplementary material for: Long-Term Evaluation and Normal Tissue Complication Probability (NTCP) Models for Predicting Radiation-Induced Optic Neuropathy after Intensity-Modulated Radiation Therapy (IMRT) for Nasopharyngeal Carcinoma: A Large Retrospective Study in China
Source: J Oncol. 2022 Feb 23;2022:3647462. doi: 10.1155/2022/3647462 (PMC8890878; doi:10.1155/2022/3647462)
Supplement: Supplementary Materials — The supplementary document includes a summary, supplementary methods and results, supplementary figures, supplementary tables, and supplementary R codes. Among them, supplementary methods and results include patients restaging, evaluations before diagnosis and treatment, models for selecting significant dosimetry predictors (RFE, CoxBoost, RF-SRC, and LASSO), and the evolution of visual acuity and only show the R codes of the RFE, CoxBoost, RF-SRC, and LASSO for selection of important dosimetric variables. Supplementary Figure 1. Flowchart of patient selection. Abbreviations: NPC = nasopharyngeal carcinoma; IMRT = intensity-modulated radiotherapy; Dmax = maximum point dose; RT = radiation therapy; LASSO = the least absolute shrinkage and selection operator; AUC = area under the receiver operating characteristic curve. Supplementary Figure 2. Pre-IMRT and post-IMRT for best-corrected visual acuity disease distribution are shown for the left and right eyes on a 5-grade scale. Abbreviations: IMRT = intensity-modulated radiation therapy. Supplementary Figure 3. An example of RION blindness (both eyes). (A) An advanced NPC invading the paraorbital regions (green arrow) and a patient with diplopia at the start of therapy; (B) IC + CCRT treatment: IC was TPF regimen for 3 cycles, CC was cisplatin for 2 cycles, and a scheduled total dose of 70.08 Gy (EQD2Gy = 73.78 Gy) in 32 fractions with the OC and ONs was included in the radiation field with a Dmax of the OC of 78.52 Gy (EQD2Gy = 82.66 Gy); and (C) 24 months after IMRT, both eyes presented acute, painless, progressive, and irreversible loss of vision within four months, and the OC was enhanced in T1WI + C (red arrow). TPF (cisplatin (60 mg/m2) with 5-fluorouracil (600 mg/m2 over 120 h) and docetaxel (60 mg/m2)); cisplatin (100 mg/m2) given in weeks 1 and 4 of radiotherapy. Abbreviations: RION = radiation-induced optic neuropathy; RT = radiation therapy, IC = induction chemotherapy, CC = concurrent chemotherapy; Dmax = ma [file 3647462.f1.pdf]

INDEX OF SUPPLEMENTARY INFORMATION

SUMMARY……….......……..…......……..…......…….............……..…………..…Page 2

SUPPLEMENTARY METHODS AND RESULTS ..……………..………. Page 3

SUPPLEMENTARY FIGURES ……..…………….……..……......…….….……Page 9

SUPPLEMENTARY TABLES………..……..……………….………….……..….Page 23

SUPPLEMENTARY R CODES…………………………………………… Page 46

SUMMARY

Radiation-induced optic neuropathy (RION) after intensity-modulated radiation therapy (IMRT) is not well-expounded. In our retrospective study, the incidence of RION remained low after IMRT (5-year incidence =1.19%). Dmax to OC and ONs should always follow the ALARP (As Low As Reasonably Practicable) principles. In our study, Dmax < 60 Gy is safe and represents an acceptable dose constraint for most NPC patients receiving IMRT. A reasonable trade-off for selected patients with unsatisfactory tumor coverage due to proximity to the optic apparatus would be Dmax < 65 Gy. Clinical risk factors included age, advanced T stage, tumor infiltration/compression of the optic nerve/chiasm. Our nomogram shows strong efficacy in predicting RION, allowing for individualized follow-up.

SUPPLEMENTARY METHODS AND RESULTS

Patients restaging

All of the 3,662 patients were restaged by two experienced radiation oncologists specializing in head and neck cancer according to the 8th edition of the AJCC/UICC staging system, mainly based on radiological imaging, and radiologic reports were taken as reference; disagreements were resolved by consensus.

Evaluations before diagnosis and treatment

Before diagnosis and treatment, eligible patients underwent a series of evaluations, including physical examinations, complete blood count with differential count, biochemical profile, plasma EBV DNA, EBV serology at baseline, head and neck magnetic resonance imaging (MRI) with contrast, nasopharyngoscopy, and electrocardiography. Either chest radiograph/computed tomography (CT) with contrast, abdominal sonography/CT with contrast, and bone scans or positron emission tomography computed tomography (PET/CT) were used to assess distant metastasis.

Models for selecting significant dosimetry predictors

Recursive feature elimination (RFE)

Recursive Feature Elimination (RFE) is a wrapper-type feature selection algorithm, which implements backwards selection of predictors based on predictor importance ranking. We first trained the survival model with the right censored time on all sets of variables, the variable importance score was obtained using the permutation method, and the least important variable was removed. The model was refitted, and evaluated on the out-of-bag samples. The above steps were repeated until all features were traversed. The optimal subset of variables were selected by the error rate for the out-of-bag samples. For this method, R packages mlr3, mlr3learners, mlr3proba, mlr3fselect, and mlr3tuning were used. The selected variables were Dmax = 0.0679809876409885, and D50 = 0.0500034216834.

Boosting in Cox regression (CoxBoost)

Boosting in Cox regression (CoxBoost) analyses is used to fit a Cox proportional hazards model by componentwise likelihood based boosting. It is especially suited for models with a large number of predictors and allows for mandatory covariates with unpenalized parameter estimates[1-2]. For the entire cohort, we proposed a weighted regression approach using a componentwise likelihood-based boosting method which was implemented in the R package

CoxBoost (https://github.com/binderh/CoxBoost). Optional covariates with non-zero coefficients at the boosting step 500 were: parameter estimates > 0: Dmin, Dmax, D25, D35, D50, parameter estimates < 0: Dmean, D0.07cc, D0.09cc (Supplementary Fig. 4).

Random Forests for Survival, Regression and Classification (RF-SRC)

Random forest (RF) is an ensemble bagged method, which grows binary classification trees based on bootstrapping the data by a random subset of available features at each node to figure out an appropriate splitting rule[3]. After repetition, RF generates numerical decorrelated decision trees that provide more robust committee-type decisions. Each tree offers a classification, and the forest chooses the one with the most votes. RandomforestSRC: Random Forests for Survival, Regression and Classification (RF-SRC). A unified treatment of Breiman's random forests for survival, regression and classification problems were based on Ishwaran and Kogalur's random survival forests (RSF) package. The package runs in both serial and parallel (OpenMP) modes. Now extended to include multivariate and unsupervised forests[4-6]. RF-SRC has been proposed as an alternative to traditional survival models, as it is able to overcome most of the limitations of traditional survival techniques, such as Cox proportional hazards models (Supplementary Fig. 5).

Least absolute shrinkage and selection operator (LASSO)

For the entire cohort, we used the R package glmnet to perform LASSO for the survival regression model with 20 dosimetry parameters of the OC/ONs (parameters in Supplementary Table 3, except volume). Cross-validation methods (cv.glmnet) were used to test the robustness of the significant candidate variables that were chosen by the Cox's proportional hazard (PH) models, in which Harrel's concordance (C-index) was used to measure the loss function. This produced a plot and a return value for lambda and gamma, which showed the C-index varying with the log (lambda) values to determine how many candidate variables should be chosen (Supplementary Fig. 6). According to the lambda1se (0.0432), we identified the minimum critical variables based on the entire cohort. We calculated the area under the curve (AUC) of the time-dependent ROC with censored time for each single selected variable and obtained the corresponding cutoff values and the corresponding sensitivities and specificities (Supplementary Table 5). Univariate analyses were applied using Cox PH regression analysis, in which the hazard ratio (HR) and 95% confidence intervals (95% CI) were calculated to compare the risk between high and low risk groups.

The evolution of visual acuity

Pre-IMRT patients who had “no visual acuity deficiency” and those with “visual acuity deficient diseases” for the left eye were in 3,485 (95.2%) and 177 (4.8%), respectively. For post-IMRT patients they were 3,265 (89.2%) and 397 (10.8%), respectively. For the Pre-IMRT patients who had “no visual acuity deficiency” and those with “visual acuity deficient diseases” for the right eye were 3,481 (95.1%) and 181 (4.9%), respectively. For post-IMRT patients they were 3,262 (89.1%) and 400 (10.9%), respectively.

Visual acuity deficient diseases were classified as those that affected and unaffected BCVA eye diseases. For example, patients that had ophthalmological diseases, such as ocular motility disorder, ptosis, dry eyes, or epiphora, if it did not affect their BCVA, they were classified as grade 1. Grade 1-5 pre-IMRT BCVA grades for the left eye were 168 (4.6%), 6 (0.2%), 3 (0.1%), 0 and 0, respectively. Post-IMRT they were 315 (8.6%), 44 (1.2%), 2 (0.1%), 10 (0.3%) and 26 (0.7%), respectively. Grade 1-5 pre-IMRT BCVA grades for the right eye were 162 (4.4%), 14 (0.4%), 3 (0.1%), 2 (0.1%) and 0, respectively. Post-IMRT they were 317 (8.7%), 34 (0.9%), 7 (0.2%), 7 (0.2%) and 35 (1%), respectively. For the BCVA grades 1-5 for visual acuity deficient disease distribution pre-IMRT and post-IMRT see Supplementary Fig.2.

REFERENCES

[1] Weyer, V., & Binder, H.. A weighting approach for judging the effect of patient strata on high-dimensional risk prediction signatures. BMC Bioinformatics. 2015 Sep 15;16:294.

[2] Binder, H (2013).Coxboost: cox models by likelihood based boosting for a single survival endpoint or competing risks. 2013.

[3] Leo Breiman. Random Forests. Mach Learn 2001;45:5-32.

[4] Ishwaran H, Kogalur U. Random survival forests for R. R News. 2007; 7(2):25-31.

[5] Mogensen UB, Ishwaran H, Gerds TA. Evaluating random forests for survival analysis using prediction error curves. J Stat Softw. 2012 Sep;50(11):1-23.

[6] Ishwaran H, Kogalur U (2021). Fast Unified Random Forests for Survival, Regression, and Classification (RF-SRC). R packageversion 2.12.0, https://cran.r-project.org/package = randomForestSRC.

SUPPLEMENTARY FIGURES

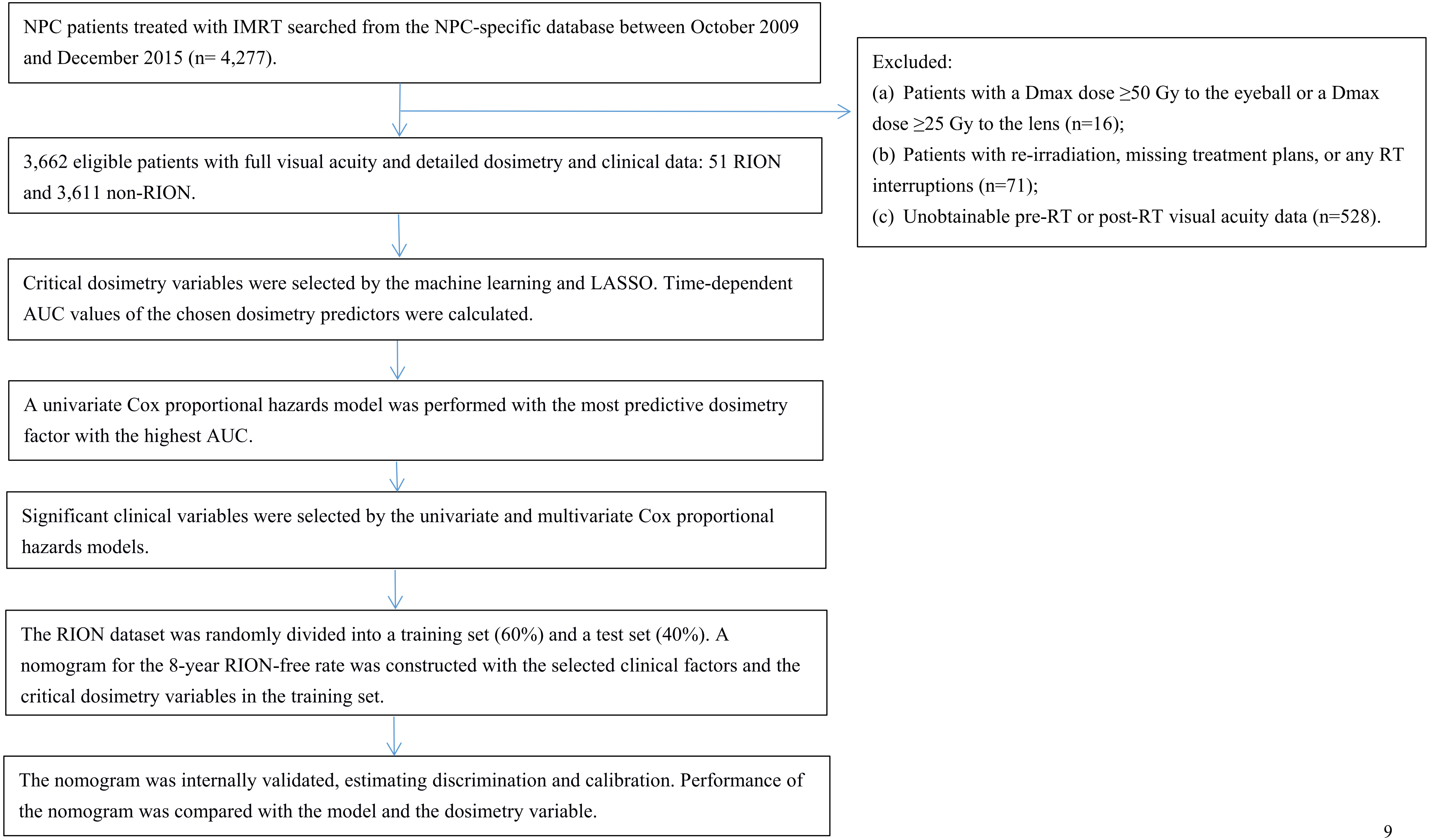

Supplementary Fig. 1. Flowchart of patient selection. Abbreviations: NPC= nasopharyngeal carcinoma; IMRT= intensity modulated radiotherapy; Dmax = maximum point dose; RT=radiation therapy; LASSO= the least absolute shrinkage and selection operator; AUC= area under the receiver operating characteristic curve.

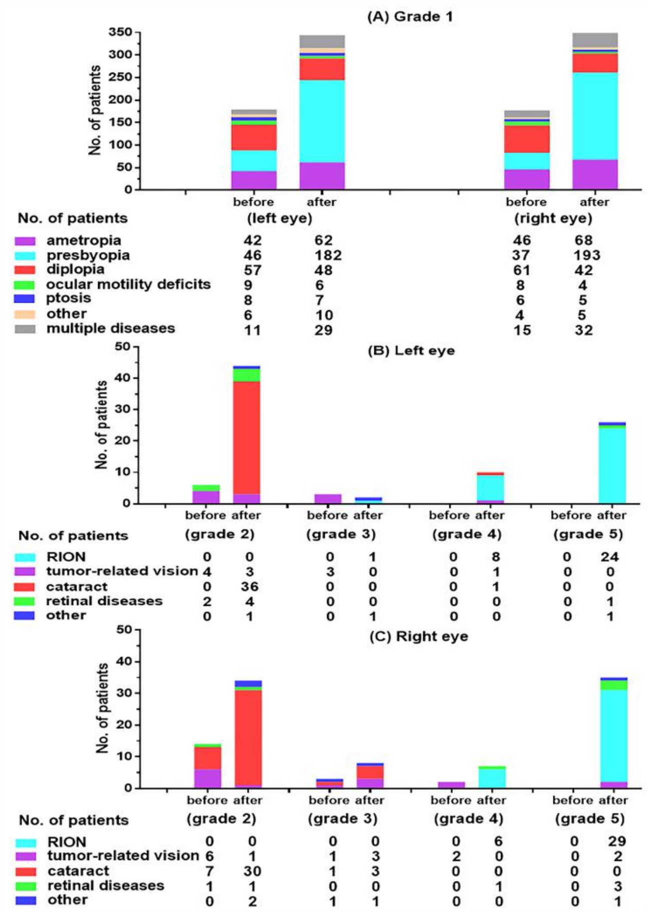

Supplementary Fig. 2. Pre-IMRT and post-IMRT for best-corrected visual acuity disease distribution are shown for the left and right eyes on a 5-grade scale.

Abbreviations: IMRT = intensity-modulated radiation therapy.

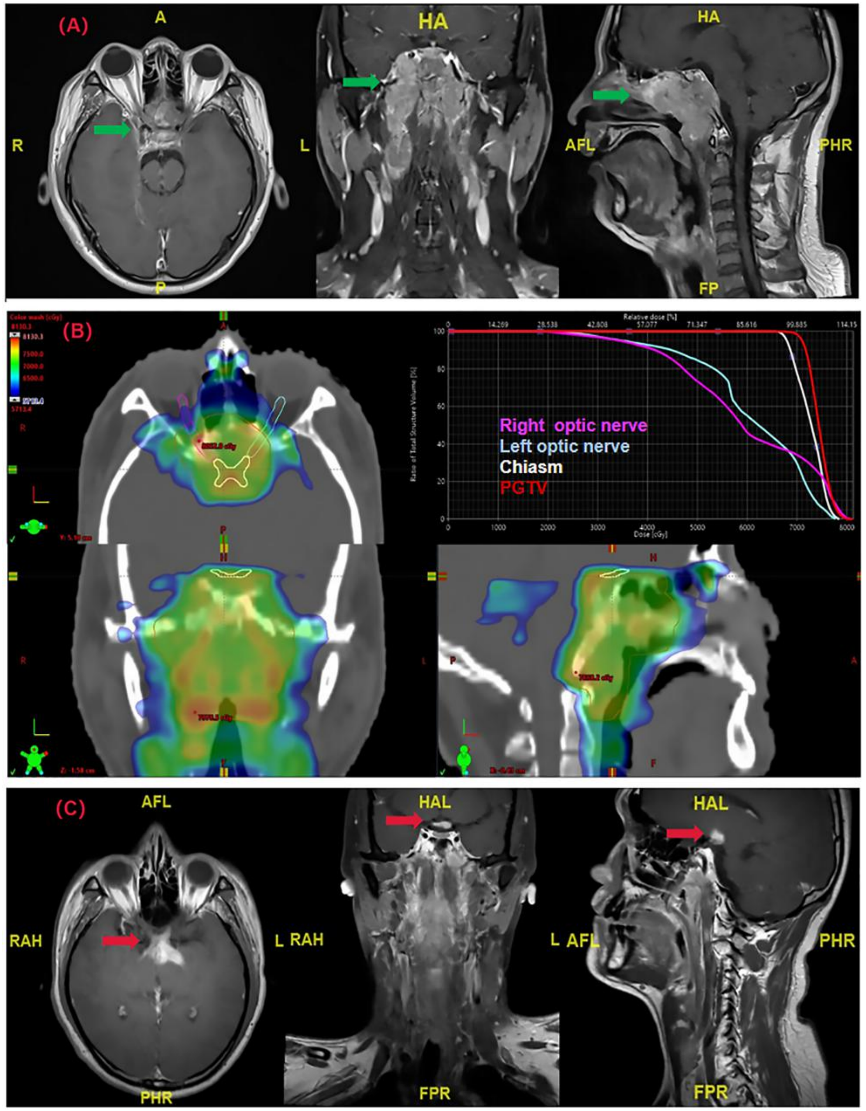
(A)
A
R
P
HA
L
HA
AFL
PHR
FP
(B)
Right optic nerve
Left optic nerve
Chiasm
PGTV
(C)
AFL
RAH
L
PHR
HAL
RAH
FPR
HAL
L
AFL
PHR
FPR

Supplementary Fig. 3. An example of RION blindness (both eyes). (A) an advanced NPC invading the paraorbital regions (green arrow) and a patient with diplopia at the start of therapy; (B) IC+CCRT treatment: IC was TPF regimen for 3 cycles and CC was cisplatin for 2 cycles, a scheduled total dose of 70.08Gy (EQD_{2Gy} =73.78 Gy) in 32 fractions with the OC and ONs included in the radiation field with a Dmax of the OC of 78.52 Gy (EQD_{2Gy} =82.66 Gy); (C) 24 months after IMRT, both eyes presented acute, painless, progressive and irreversible loss of vision within four months, the OC was enhanced in T1WI+C (red arrow). TPF [cisplatin (60mg/m^2) with 5-fluorouracil (600mg/m^2 over 120 h), and docetaxel (60mg/m^2)]；cisplatin (100mg/m^2) given in weeks 1 and 4 of radiotherapy. Abbreviations: RION = radiation-induced optic neuropathy; RT = radiation therapy, IC = induction chemotherapy, CC = concurrent chemotherapy; Dmax = maximum point dose; OC = optic chiasma; ONs = optic nerves; T1WI +C = postcontrast T1-weighted image.

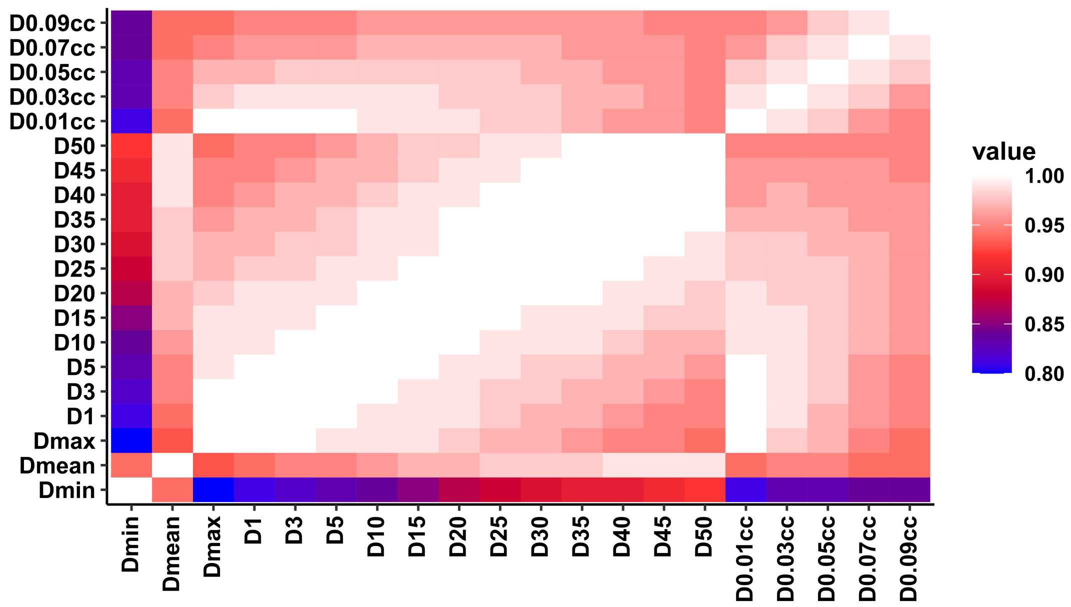

Supplementary Fig. 4. Spearman's correlation matrix of 20 dosimetry factors. Value means Spearman's correlation coefficient. It shows strong correlation with dosimetry factors. Dmin = minimum point dose; Dmean = mean dose; Dmax = maximum point dose; D1-50 = minimum dose 1%-50% volume of the optic nerve or optic chiasma; D0.01-0.09cc = minimum dose to 0.01-0.09 cc volume of the optic nerve or optic chiasma.

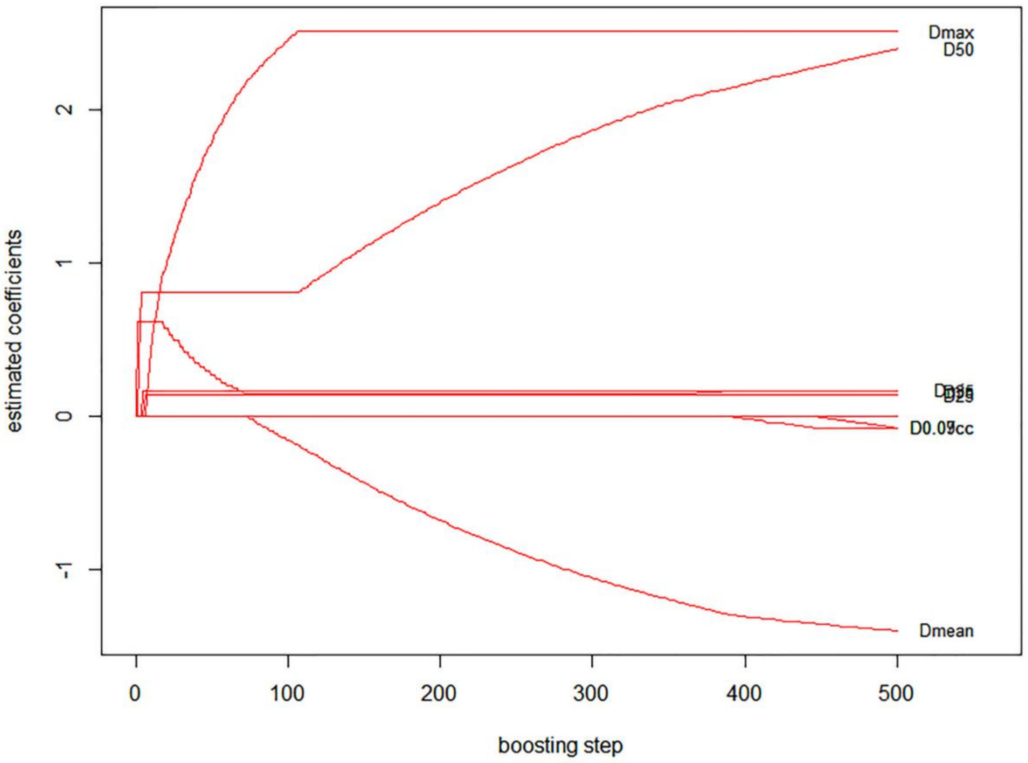

Supplementary Fig. 5. Coefficient paths for the 20 dosimetry factors using boosting in cox regression. Optional covariates with non-zero coefficients at boosting step 500: parameter estimate > 0: Dmin, Dmax, D25, D35, D50, parameter estimate < 0: Dmean, D0.07cc, D0.09cc. Dmin = minimum point dose; Dmean = mean dose; Dmax = maximum point dose; D1-50 = minimum dose 1%-50% volume of the optic nerve or optic chiasma; D0.01-0.09cc = minimum dose to 0.01-0.09 cc volume of the optic nerve or optic chiasma.

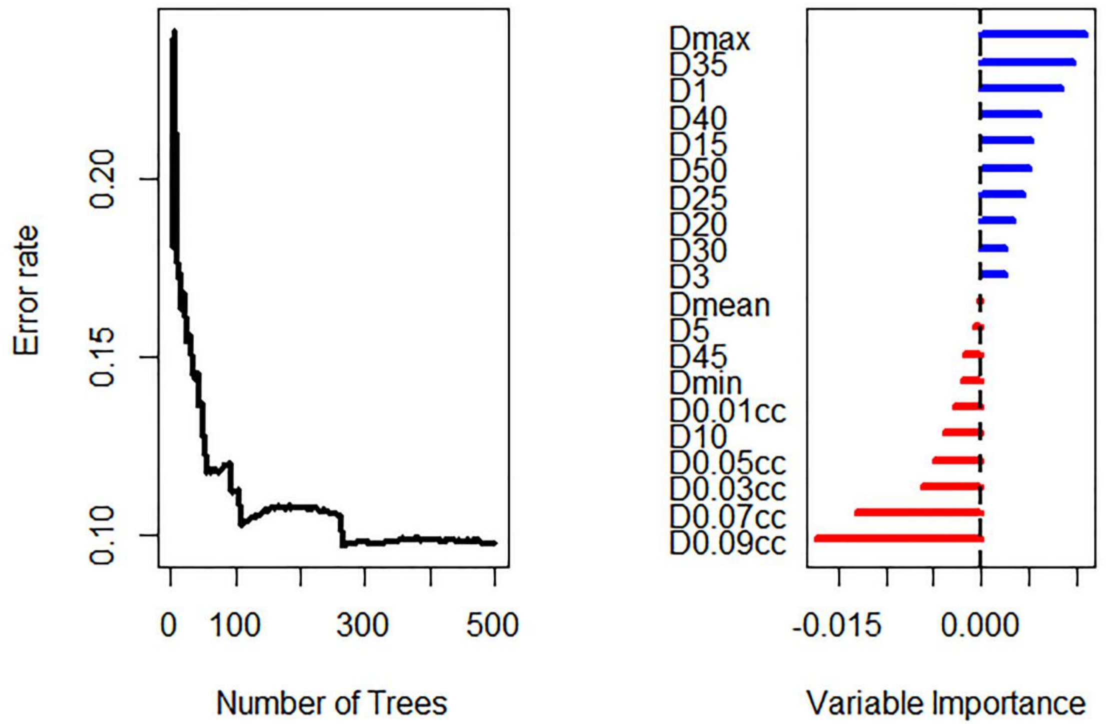

Supplementary Fig. 6. Important variables selected from the 20 dosimetry factors using the random survival forest model. Dmin = minimum point dose; Dmean = mean dose; Dmax = maximum point dose; D1-50 = minimum dose 1%-50% volume of the optic nerve or optic chiasma; D0.01-0.09cc = minimum dose to 0.01-0.09 cc volume of the optic nerve or optic chiasma.

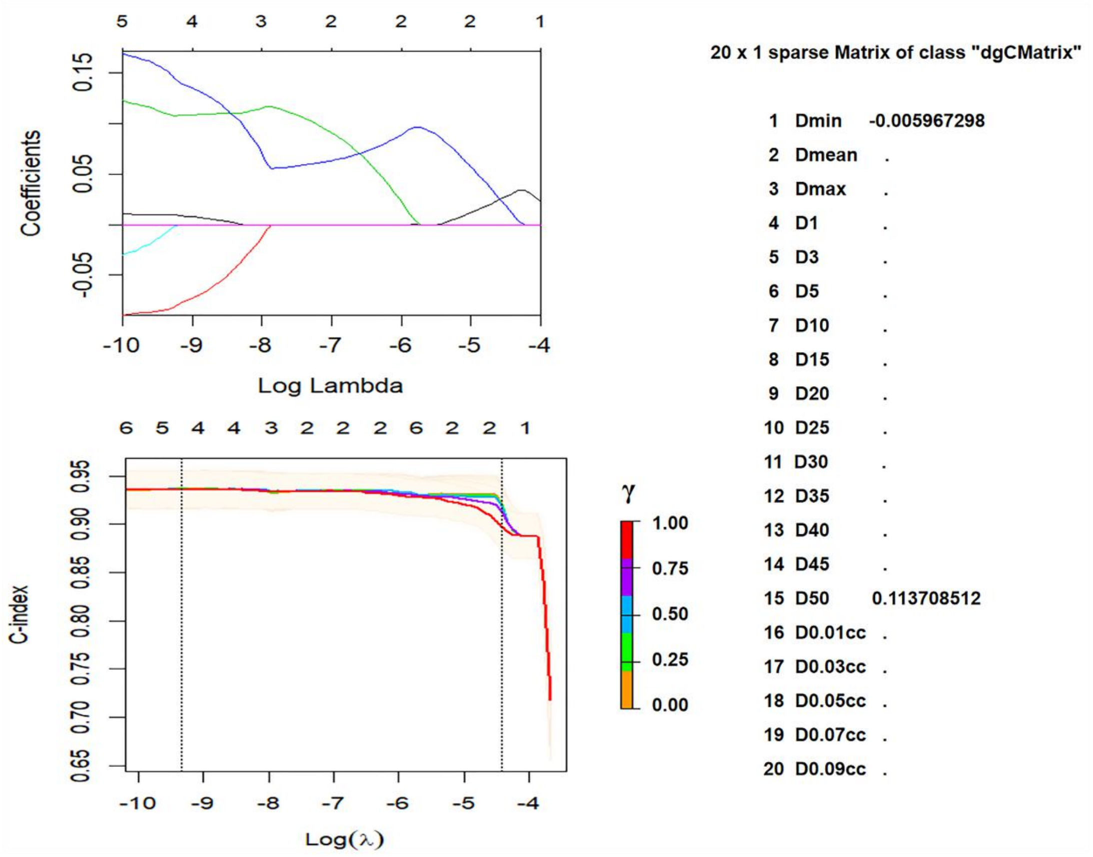

Supplementary Fig. 7. Plot of LASSO coefficients and selective variables at lambda.1se (0.0432). LASSO=Least Absolute Shrinkage and Selection Operator. Dmin = minimum point dose; Dmean = mean dose; Dmax = maximum point dose; D1-50 = minimum dose 1%-50% volume of the optic nerve or optic chiasma; D0.01-0.09cc = minimum dose to 0.01-0.09 cc volume of the optic nerve or optic chiasma.

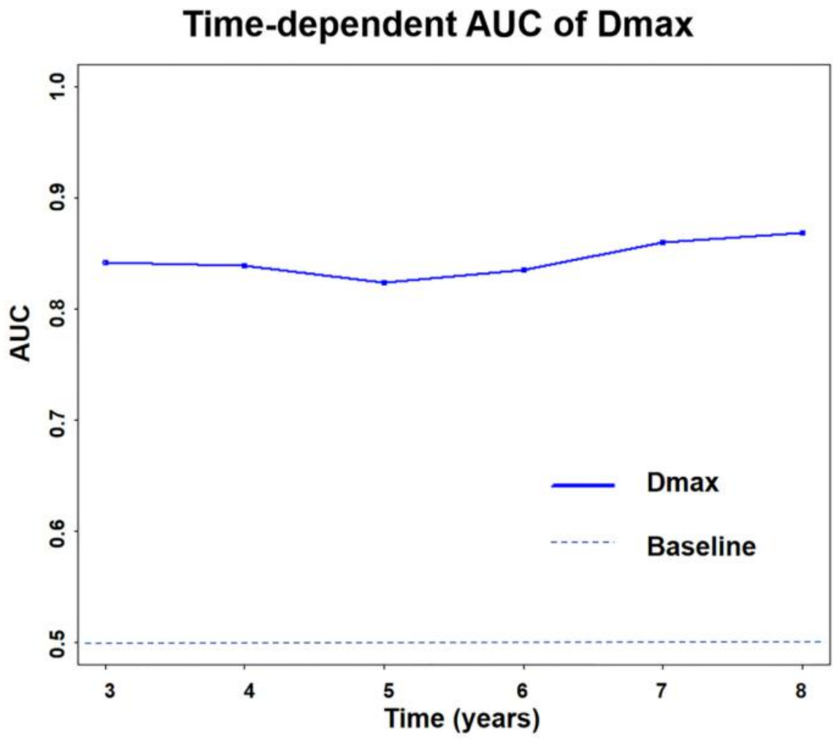

Supplementary Fig. 8. The lines depict the time-dependent AUC for Dmax. Abbreviations: AUC = area under the receiver operating characteristic curve.

Dmax = maximum point dose.

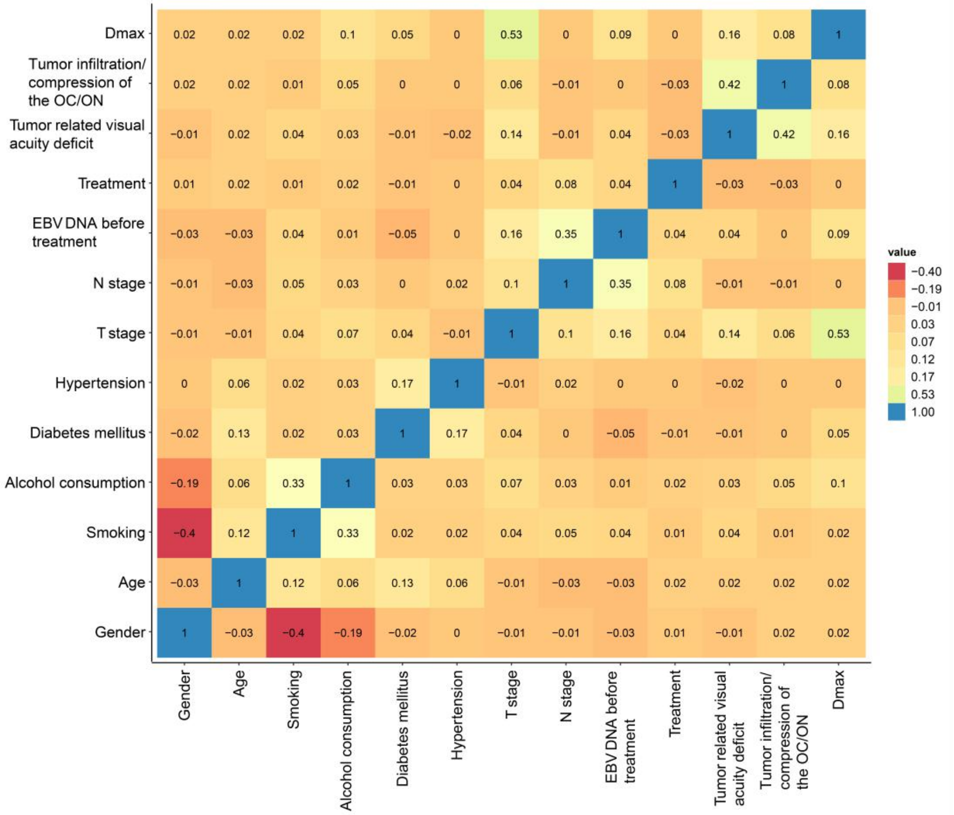

Supplementary Fig. 9. Spearman's correlation matrix between significant clinical variables and the most important dosimetry predictor: Dmax. Value means Spearman's correlation coefficient. There were low-to-moderate correlations between Dmax and the significant clinical variables, with the highest Spearman coefficient: 0.53. Abbreviations: Dmax= maximum point dose; OC=optic chiasma; ON=optic nerve; EBV =Epstein-Barr virus.

	aHR (95% CI)	P Value
Univariate		
Age, years		
≥ 44 vs. <44	2.175 (1.204, 3.931)	0.01
Gender		
Female vs. Male	1.563 (0.880, 2.776)	0.127
Smoking		
Yes vs. No	1.115 (0.628, 1.980)	0.711
Alcohol consumption		
Yes vs. No	1.166 (0.584, 2.327)	0.663
Comorbidities		
Diabetes mellitus		
Yes vs. No	1.378 (0.429, 4.426)	0.59
Hypertension		
Yes vs. No	1.987 (0.790, 5.001)	0.145
Tumor−related visual acuity deficient before therapy		
Yes vs. No	8.834 (4.423, 17.643)	< 0.001
Tumor infiltration/compression of the chiasm/optic nerves		
Yes vs. No	24.048 (9.553, 60.536)	< 0.001
EBV DNA before treatment, cogy/ml		
≥ 2,000 vs.<2,000	1.914 (1.096, 3.343)	0.023
T stage		
T1, T2	Reference	
T3	7.901 (1.816, 34.364)	0.006
T4	46.223 (11.090, 192.661)	< 0.001
N stage		
N0	Reference	
N1	1.149 (0.493, 2.677)	0.748
N2	2.029 (0.834, 4.937)	0.119
N3	1.172 (0.372, 3.697)	0.786
Treatment...		
RT alone	Reference	
IC+CCRT group	2.576 (0.905, 7.329)	0.076
CCRT group	1.306 (0.442, 3.858)	0.629
Multivariate		
Age, years		
≥ 44 vs. <44	2.234 (1.233, 4.051)	0.008
Tumor−related visual acuity deficient before therapy		
Yes vs. No	2.199 (0.854, 5.669)	0.103
Tumor infiltration/compression of the chiasm/optic nerves		
Yes vs. No	4.572 (1.316, 15.874)	0.017
EBV DNA before treatment, cogy/ml		
≥ 2,000 vs.<2,000	1.311 (0.746, 2.305)	0.346
T stage		
T1, T2	Reference	
T3	7.516 (1.725, 32.767)	0.007
T4	37.189 (8.796, 157.266)	< 0.001

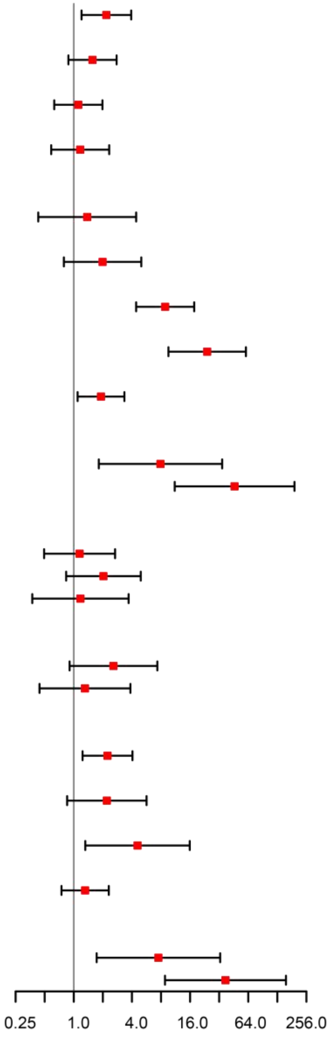

Supplementary Fig. 10. Forest plots depicting the univariate and multivariate associations of clinical factors with RION. Squares represent aHRs with 95% CIs indicated by the horizontal bars. The treatment was a combination of radiotherapy and chemotherapy, IC+CC group = IC ± CC ± AC ± target; CC group = CC ± AC ± target. Abbreviations: RION = radiation-induced optic neuropathy; aHR = adjusted hazard ratio; CIs = confidence intervals; EBV = Epstein-Barr virus; RT = radiation therapy, IC = Induction chemotherapy, CC = concurrent chemotherapy, AC = adjuvant chemotherapy.

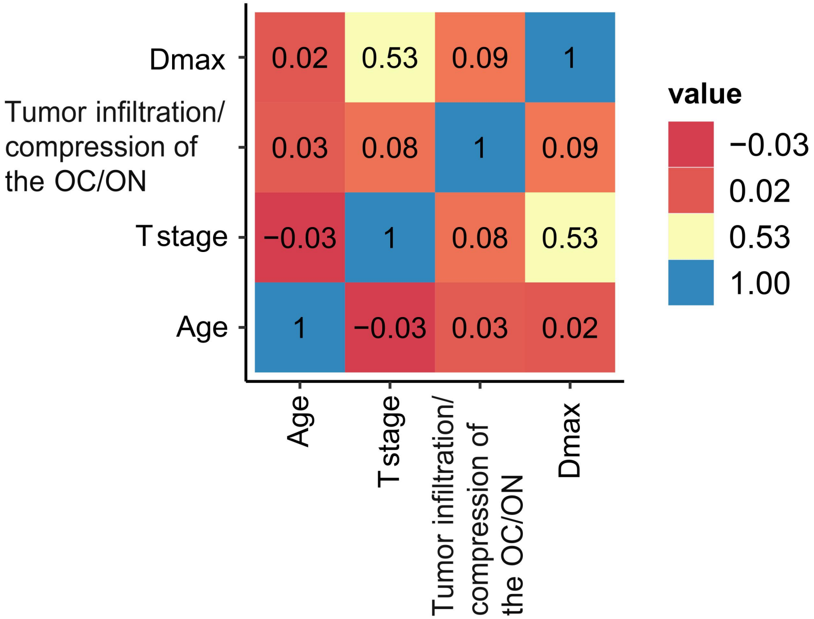

Supplementary Fig. 11. Spearman's correlation matrix between significant clinical variables and Dmax. Value means Spearman's correlation coefficient. There were low-to-moderate correlations between Dmax and the significant clinical variables, with the highest Spearman coefficient: 0.53. Abbreviations: Dmax= maximum point dose; OC=optic chiasma; ON=optic nerve.

SUPPLEMENTARY TABLES

Supplementary Table 1

Details on patients with decreased visual acuity associated with RION.

Pat. -No.	Age (yr.)	Baseline clinical risk factors	Baseline eye disorders	Treatment	The highest Dmax (EQD_{2Gy}) for OC or ON	Latent time (months)	Decrease in acuity pre-IMRT→post-IMRT (grades)	Symptoms
**1**	45	Alcohol	None	IC	83.19 (chiasm)	90	1→5 (right side)	Painless, progressive and irreversible loss of vision.
**2**	28	No	Double images	IC+CC	82.66 (chiasm)	24	1→blind (both side)	Painless, progressive and irreversible loss of vision.
**3**	39	No	None	RT alone	82.59 (chiasm)	84	1→4 (left side)	Painless, progressive and irreversible loss of vision.

**4**	54	Smoking	Double images	IC+CC+target	82.49 (chiasm)	24	1→blind (left side)	Painless, progressive and irreversible loss of vision.
**5**	49	No	None	IC+CC	82.43 (right optic nerve)	24	1→blind (both side)	Painless, progressive and irreversible loss of vision.
**6**	25	No	None	IC+CC	81.83 (left optic nerve)	48	1→5 (left side)	Temporal field loss
**7**	45	Alcohol	Blurred vision (left side)	IC+CC+AC	81.35 (chiasm)	36	2→blind (left side) 1→5 (right side)	Painless, progressive and irreversible loss of vision.
**8**	45	No	Blurred vision (right side)	IC+CC	81.33 (right optic nerve)	12	2→blind (right side)	Painless, progressive and irreversible loss of vision.
**9**	34	Smoking	Double images	IC+CC	81.13 (right optic nerve)	13	1→blind (right side)	Painless, progressive and irreversible loss of vision; Double images.

**10**	43	Smoking; Alcohol.	Double images	IC+CC	80.78 (right optic nerve)	36	1→blind (right side)	Painless, progressive and irreversible loss of vision.
**11**	50	Smoking	None	IC	80.27 (chiasm)	48	1→blind (left side)	Painless, progressive and irreversible loss of vision.
**12**	32	Smoking	Blurred vision (left side)	IC+CC	80.24 (left optic nerve)	3	3→blind (left side)	Loss of vision (left side) due to tumor optic nerve compression, blind three months after the completion of IMRT.
**13**	55	Smoking; Hypertension.	None	IC+CC	80.24 (left optic nerve)	80	1→5 (left side)<br>1→4 (right side)	Painless, progressive and irreversible loss of vision.
**14**	58	Alcohol; Hypertension.	Blurred vision (right side)	IC+CC	80.2 (chiasm)	76	1→4 (left side)<br>2→blind (right side)	Painless, progressive and irreversible loss of vision

								(right side); cataract (left side).
**15**	39	Smoking	None	RT alone	79.97 (chiasm)	36	1→5 (right side)	Painless, progressive and irreversible loss of vision.
**16**	55	Smoking	None	IC+CC	79.97 (chiasm)	24	1→4 (right side)	Painless, progressive and irreversible loss of vision; cataract.
**17**	35	No	None	IC	79.79 (chiasm)	12	1→5 (left side)<br>1→blind (right side)	Painless, progressive and irreversible loss of vision.
**18**	44	No	None	CC	79.63 (chiasm)	24	1→5 (right side)	Painless, progressive and irreversible loss of vision.
**19**	39	No	None	IC+CC	79.11 (chiasm)	60	1→4 (right side)	Painless, progressive and irreversible loss of vision.

**20**	63	Smoking; Alcohol.	None	IC+CC	78.97 (chiasm)	3	1→4 (left side) 1→blind (right side)	Painless, progressive and irreversible loss of vision.
**21**	58	No	Ocular motility disorder; double images; blurred vision (right side).	IC+CC	78.89 (chiasm)	12	1→5 (left side) 3→blind (right side)	Painless, progressive and irreversible loss of vision (both sides); ocular motility disorder (right side).
**22**	55	No	None	IC+CC	78.84 (chiasm)	30	1→blind (both sides)	Painless, progressive and irreversible loss of vision.
**23**	22	No	None	IC+CC	78.8 (left optic nerve)	36	1→blind (both sides)	Painless, progressive and irreversible loss of vision.
**24**	58	Smoking	None	CC	78.25 (chiasm)	80	1→5(left side)	Painless, progressive and irreversible loss of vision.

**25**	43	Smoking	None	IC	78.21 (chiasm)	72	1→4 (right side)	Painless, progressive and irreversible loss of vision.
**26**	52	Alcohol	None	CC	77.99 (chiasm)	48	1→4 (left side)	Painless, progressive and irreversible loss of vision.
**27**	48	No	None	CC	77.49 (chiasm)	36	1→5 (right side)	Painless, progressive and irreversible loss of vision.
**28**	38	No	None	CC	76.8 (chiasm)	72	1→blind (right side)	Painless, progressive and irreversible loss of vision.
**29**	64	No	None	RT alone	76.46 (chiasm)	35	1→5 (right side)	Painless, progressive and irreversible loss of vision.
**30**	50	Smoking	None	IC+CC	76.38 (chiasm)	70	1→4 (left side)	Painless, progressive and irreversible loss of visi on; ocular motility disor

								der; double images.
**31**	66	Smoking; DM.	None	IC+CC+target	75.67 (chiasm)	50	1→blind (both sides)	Painless, progressive and irreversible loss of vision.
**32**	51	Alcohol	None	IC+CC	75.26 (chiasm)	36	1→blind (left side)	Painless, progressive and irreversible loss of vision.
**33**	51	Smoking	None	IC	73.84 (chiasm)	58	1→5 (left side) 1→blind (right side)	Painless, progressive and irreversible loss of vision.
**34**	54	Smoking	None	CC	73.61 (chiasm)	70	1→5 (left side) 1→blind (right side)	Painless, progressive and irreversible loss of vision.
**35**	22	No	None	IC+CC	73.38 (left optic nerve)	36	1→4 (left side)	painless, progressive and irreversible loss of vision.
**36**	59	No	None	CC+AC	72.83 (chiasm)	60	1→5 (left side)	Painless, progressive and irreversible loss of vision.

**37**	34	No	None	CC	72.8 (chiasm)	36	1→blind (right side)	Painless, progressive and irreversible loss of vision.
**38**	56	Smoking	None	IC+CC	72.69 (left optic nerve)	12	1→5 (right side)	Painless, progressive and irreversible loss of vision.
**39**	32	Smoking; Alcohol.	None	CC	72.63 (chiasm)	48	1→4 (left side)	Painless, progressive and irreversible loss of vision.
**40**	55	No	None	IC+CC	72.26 (left optic nerve)	80	1→5 (left side) 1→blind (right side)	Painless, progressive and irreversible loss of vision.
**41**	54	No	None	IC+CC	72.18 (right optic nerve)	60	1→5 (left side)	Painless, progressive and irreversible loss of vision.
**42**	73	Hypertension; DM.	presbyopia	RT alone	71.33 (chiasm)	24	1→5 (left side) 1→blind (right side)	Painless, progressive and irreversible loss of vision; presbyopia.

**43**	54	DM	Myopia	IC	68.56 (chiasm)	48	1→5 (left side)	Painless, progressive and irreversible loss of vision; double images; cataract.
**44**	61	Hypertension	None	IC+CC	67.78 (chiasm)	48	1→5 (left side) 1→4 (right side)	Painless, progressive and irreversible loss of vision.
**45**	56	Smoking; Alcohol.	None	IC+CC	67.46 (right optic nerve)	12	1→4 (left side) 1→5 (right side)	Painless, progressive and irreversible loss of vision.
**46**	46	No	Cataract	IC	67.27 (chiasm)	36	1→5 (left side)	Painless, progressive and irreversible loss of vision; cataract.
**47**	51	No	None	IC	67.19 (chiasm)	58	1→4 (left side)	Painless, progressive and irreversible loss of vision; presbyopia.

**48**	48	No	None	IC+CC+target	65.22 (chiasm)	36	1→5 (right side)	Painless, progressive and irreversible loss of vision; dry eyes.
**49**	61	Alcohol	None	IC+CC	64.6 (chiasm)	60	1→blind (right side)	Painless, progressive and irreversible loss of vision.
**50**	16	No	None	IC+CC	62.22 (chiasm)	24	1→3 (left side)<br>1→4 (right side)	Generalized constricted fields (both eyes).
**51**	59	Hypertension	None	CC	58.27 (chiasm)	36	1→5 (left side)<br>1→blind (right side)	Painless, progressive and irreversible loss of vision.

Abbreviations: RION = Radiation-induced optic neuropathy; IMRT = intensity modulated radiotherapy; Dmax = maximum point dose; RT = radiation therapy; IC = induction chemotherapy; CC = concurrent chemotherapy; AC = adjuvant chemotherapy; OC = optic chiasma; ON = optic nerve.

Supplementary Table 2

Dose-level stratifications for Dmax to the OC/ON.

	The Dmax dose in OC/ON (group)						
**Factor**	**≥70 Gy**	**65-<70Gy**	**60-<65Gy**	**55-<60Gy**	**50-<55Gy**	**<50Gy**	**Total**
No. of patients	431 (%)	267 (%)	316 (%)	311 (%)	487 (%)	1,850 (%)	3,662 (%)
the Dmax dose in OC/ON (Gy)(Mean ± SD)	75.33 ± 1.56	67.44 ± 1.49	62.62 ± 1.44	57.36 ± 1.36	52.45 ± 1.38	29.4 ± 14.8	45.9 ± 20.69
RION event							
Yes	42 (9.7)	6 (2.2)	2 (0.6)	1 (0.3)	0	0	51 (1.4)
No	389 (90.3)	261 (97.8)	314 (99.4)	310 (99.7)	487 (100)	1,850 (100)	3,611 (98.6)
Time for RION (months) Median (min–max)	36 (3-90)	36 (12-58)	24, 60	36	/	/	36 (3-90)
Symptoms with RION							
progressive loss of vision	41 (9.5)	6 (2.2)	1 (0.3)	1 (0.3)	0	0	49 (1.3)
two-sided progressive loss of vision	13 (3)	2 (0.7)	0	1 (0.3)	0	0	16 (0.4)

blind	22 (5.1)	0	1 (0.3)	1 (0.3)	0	0	24 (0.7)
two-sided blind	4 (0.9)	0	0	0	0	0	4 (0.1)
partial visual field loss	1 (0.2)	0	1 (0.3)	0	0	0	2 (0.1)
two-sided partial visual field loss	0	0	1 (0.3)	0	0	0	1 (0)
#Combined with RT-unrelated symptoms	3 (0.7)	2 (0.7)	0	0	0	0	5 (0.1)

#Combined with RT-unrelated symptoms with diplopia (n =2), exotropia (n =2), dry eyes (n =1).

It is noteworthy that the highest Dmax out of all doses to the left/right ONs and OC was used in the analysis.

Abbreviations: RION =Radiation-induced optic neuropathy; Dmax = maximum point dose; SD = standard deviation.

Supplementary Table 3

Comparison of dosimetry parameters between RION and non-RION patients.

Variable	Mean ± SD		P*
	**RION (n = 51)**	**Non-RION (n = 3,611)**	
Volume (cc)	0.50 ± 0.25	0.52 ± 0.26	0.896
Dmin (Gy)	56.28 ± 16.94	22.72 ± 17.85	< 0.001
Dmean (Gy)	66.92 ± 11.20	33.51 ± 19.57	< 0.001
**Dmax (Gy)**	**75.75 ± 5.94**	**45.48 ± 20.52**	**< 0.001**
D1 (Gy)	74.83 ± 6.16	44.05 ± 20.46	< 0.001
D3 (Gy)	74.20 ± 6.29	43.06 ± 20.39	< 0.001
D5(Gy)	73.76 ± 6.40	42.38 ± 20.34	< 0.001
D10 (Gy)	73.08 ± 6.56	41.07 ± 20.27	< 0.001
D15 (Gy)	72.55 ± 6.72	39.96 ± 20.23	< 0.001

D20 (Gy)	72.05 ± 6.92	38.90 ± 20.25	< 0.001
D25 (Gy)	71.58 ± 7.10	38.00 ± 20.23	< 0.001
D30 (Gy)	71.10 ± 7.26	37.12 ± 20.21	< 0.001
D35 (Gy)	70.64 ± 7.43	36.23 ± 20.20	< 0.001
D40 (Gy)	70.15 ± 7.61	35.41 ± 20.18	< 0.001
D45 (Gy)	69.64 ± 7.81	34.59 ± 20.16	< 0.001
D50 (Gy)	69.13 ± 8.02	33.76 ± 20.13	< 0.001
D0.01cc (Gy)	74.39 ± 6.27	43.29 ± 20.44	< 0.001
D0.03cc (Gy)	73.44 ± 6.53	41.73 ± 20.37	< 0.001
D0.05cc (Gy)	72.81 ± 6.70	40.69 ± 20.34	< 0.001
D0.07cc (Gy)	72.12 ± 6.93	39.77 ± 20.35	< 0.001
D0.09cc (Gy)	71.48 ± 7.16	38.89 ± 20.31	< 0.001

Abbreviations: RION =Radiation-induced optic neuropathy; SD = standard deviation; Dmin = minimum point dose; Dmean = mean dose; Dmax = maximum point dose; D1-50 = minimum dose 1%-50% volume of the optic nerve or optic chiasma; D0.01-0.09cc = minimum dose to 0.01-0.09 cc volume of the optic nerve or optic chiasma.

Note that the highest Dmax out of all doses to the left/right optic nerve or optic chiasma structure was used in the dosimetry analysis.

*From Student's t test.

Supplementary Table 4

Univariate analysis for the dosimetry parameters predicting the development RION.

**Variable**	**HR**	**95% CI**	**P***
Dmin (Gy)	1.09	1.07-1.10	< 0.001
Dmean (Gy)	1.13	1.10-1.16	< 0.001
**Dmax (Gy)**	**1.22**	**1.17-1.28**	**< 0.001**

D1 (Gy)	1.21	1.16-1.26	< 0.001
D3 (Gy)	1.20	1.16-1.25	< 0.001
D5(Gy)	1.20	1.15-1.25	< 0.001
D10 (Gy)	1.19	1.15-1.24	< 0.001
D15 (Gy)	1.19	1.14-1.23	< 0.001
D20 (Gy)	1.18	1.14-1.23	< 0.001
D25 (Gy)	1.18	1.14-1.22	< 0.001
D30 (Gy)	1.17	1.13-1.21	< 0.001
D35 (Gy)	1.17	1.13-1.21	< 0.001
D40 (Gy)	1.16	1.12-1.19	< 0.001
D45 (Gy)	1.16	1.12-1.19	< 0.001
D50 (Gy)	1.15	1.12-1.19	< 0.001
D0.01cc (Gy)	1.20	1.16-1.25	< 0.001

D0.03cc (Gy)	1.19	1.15-1.24	< 0.001
D0.05cc (Gy)	1.19	1.14-1.23	< 0.001
D0.07cc (Gy)	1.18	1.13-1.22	< 0.001
D0.09cc (Gy)	1.17	1.13-1.21	< 0.001

Abbreviations: RION =Radiation-induced optic neuropathy; HR =hazard ratio; CI = confidence interval; Dmin = minimum point dose; Dmean = mean dose; Dmax = maximum point dose; D1-50 = minimum dose to 1%-50% volume of the optic nerve or optic chiasma; D0.01-0.09cc = minimum dose to 0.01-0.09 cc volume of the optic nerve or optic chiasma.

Note that the highest Dmax out of all doses to the left/right optic nerve or optic chiasma structure was used in the dosimetry analysis.

*From Student's t test.

Supplementary Table 5

The time-dependent AUC and cutoff values of the selected variables.

Variable	Time	AUC	Cut-off score/EQD2Gy*	Sensitivity	Specificity
Dmax	3-years	0.9280	64.07	0.915	0.798
Dmax	5-years	0.9241	64.04	0.935	0.800
**Dmax**	**8-years**	**0.9434**	**64.48**	**0.955**	**0.814**
D1	3-years	0.9292	62.47	0.924	0.792
D1	5-years	0.9246	61.16	0.961	0.769
D1	8-years	0.9433	63.64	0.943	0.822
D35	3-years	0.9286	56.86	0.916	0.795
D35	5-years	0.9263	57.45	0.922	0.808
D35	8-years	0.9431	59.96	0.923	0.847
D50	3-years	0.9255	56.95	0.884	0.826

D50	5-years	0.9253	55.91	0.918	0.813
D50	8-years	0.9403	57.16	0.937	0.838
Dmin	3-years	0.8979	42.52	0.784	0.834
Dmin	5-years	0.9031	40.12	0.825	0.819
Dmin	8-years	0.9183	40.12	0.813	0.823

Abbreviations: AUC = area under the receiver operating characteristic curve; Dmax = maximum point dose; D1 = minimum dose to 1.0% volume of optic nerve or optic chiasma.

* EQD_{2Gy} is the total effective dose applied with 2-Gy equivalents per fraction.

Supplementary Table 6

Point assignment of the nomogram for RION in the overall analysis.

Variables and probability of RION	Score
Age, years	
< 44	0
≥ 44	16
T stage	
T1, T2	0
T3	28
T4	29
Tumor infiltration/compression of the chiasm/optic nerves	
No	0
Yes	41

Dmax (Gy)	
<64.48	0
≥64.48	100
3-year RION-free probability (%)	
60%	200
70%	192
80%	181
90%	164
95%	147
99%	109
5-year RION-free probability (%)	
40%	203
60%	190

70%	181
80%	170
90%	153
95%	136
99%	98
8-year RION-free probability (%)	
20%	205
40%	192
60%	178
70%	170
80%	159
90%	141
95%	124

99%	86

Abbreviations: RION = Radiation-induced optic neuropathy; Dmax = maximum point dose.

SUPPLEMENTARY R CODES

#Only show the code of the RFE, CoxBoost, RF-SRC and LASSO for selection of important dosimetric variables.

```
#The packages needed
library(glmnet)
library("survivalROC")
library("ggplot2")
library('readxl')
library("xlsx")
library("randomForestSRC")
library("CoxBoost")
library(data.table)
library(mlr3)
library(mlr3learners)
library(mlr3proba)
library(mlr3fselect)
library(mlr3tuning)
library(paradox)
library(survival)
library(gtsummary)
library(sass)
library(gtsummary)

library(readxl)
```

```
setwd("D:/data/RION data")
DRS<- read_xlsx("D:/data/RION data/data1.xlsx")
dput(names(DRS))
```

LASSO

```
library(glmnet)
data1.outcome<-cbind(time= DRS$futime,status= DRS$fustat)
data1.X<-as.matrix(DRS[,5:24])
set.seed(2000)
fit0<-cv.glmnet(data1.X,data1.outcome, family = "cox", type.measure = "C", relax = TRUE)
# type.measure="C" is Harrel's concordance measure, only available for cox models
log(fit0$lambda)
  tiff(filename = "log lambda plot0712.tiff")    #save tiff figure at local file
par(mfrow=c(1, 1), pin = c(8,4.6), cex=1.5, cex.axis=1, cex.main=1.2, font=1, font.axis=1.2)
#CV plot for lambda
plot(fit0, xvar = "lambda", sign.lambda=-1, label=TRUE)
dev.off()
fit0$lambda.min
log(fit0$lambda.min)
fit0$lambda.1se
fit0.coef.lamda.se<-coef(fit0,s=fit0$lambda.1se)
fit0.coef.lamda.se
### lasso coef plot
fit0.se.out<-fit0.coef.lamda.se[which(fit0.coef.lamda.se!=0),]
fit0.se.out<-round(fit0.se.out,4)
```

```
fit01<-glmnet(data1.X,data1.outcome,family="cox", type.measure = "C",relax=TRUE,maxp=17)
fit01
tiff(filename = "coef plot0712.tiff")
plot(fit01, xvar = "lambda",
label = TRUE,
        xlim=c(-10,-4),
        xaxs = "i", yaxs ="i",
        cex.axis=1.2,
        cex.lab=1.2,
        font.axis=1)
dev.off()
```

RF-SRC

```
# install.packages("randomForestSRC")
library("survivalROC")
library("ggplot2")
library('readxl')
library("xlsx")
library("randomForestSRC")
rf.model <- rfsrc(Surv(futime, fustat) ~ Dmin + Dmean + Dmax + D1 + D3 + D5 + D10 + D15 + D20 + D25 + D30 + D35 + D40 + D45 + D50 +    D0.01cc
+ D0.03cc + D0.05cc + D0.07cc + D0.09cc, data = DRS, nsplit = 10,    ntree=500, block.size=1,seed = 12345, importance = TRUE)
summary(rf.model)
tiff(filename = "randomForestSRC.tiff")
plot(rf.model)
dev.off()
```

CoxBoost

```
library("CoxBoost")
vars<-DRS[,c("Dmin", "Dmean", "Dmax", "D1", "D3", "D5",
            "D10", "D15", "D20", "D25", "D30", "D35", "D40", "D45", "D50",
            "D0.01cc", "D0.03cc", "D0.05cc", "D0.07cc", "D0.09cc")]
coxboost1<-CoxBoost(time=DRS$futime,status=DRS$fustat,x=as.matrix(vars),
           standardize=TRUE,
           # weights=NULL,
           stepno=500,
           penalty=100,
           # penalty=9*sum(status[subset]==1),
           # criterion = c("pscore", "score","hpscore","hscore"),
           criterion ="pscore",
           stepsize.factor=1,
           # sf.scheme=c("sigmoid","linear"),
           sf.scheme="sigmoid",
           # pendistmat=NULL,
           # connected.index=NULL,
           # x.is.01=FALSE,
           return.score=TRUE,
           trace=FALSE)
summary(coxboost1)
coef(coxboost1,as.step=500,scaled=TRUE)
tiff(filename = "CoxBoost.tiff")
```

```
plot(coxboost1,line.col="red",label.cex=0.8)
dev.off()
```

RFE

```
# install.packages("caret")
rm(list = ls())
library(readxl)
library(data.table)
library(mlr3)
library(mlr3learners)
library(mlr3proba)
library(mlr3fselect)
library(mlr3tuning)
library(paradox)
library(survival)
library(gtsummary)
library(sass)

dat <- read_xlsx("D:/data/RION data/data1.xlsx")
dat<- dat[, -c(1,4)] #delete column No. 1 and 4
##### caret rfe,e backwards selection, a.k.a. recursive feature elimination (RFE)
rfs.task = TaskSurv$new(id ="feature_selection",backend = dat,time ="futime",event = "fustat",type ="right")
rfs.learner = lrn("surv.ranger",importance ="permutation")
set.seed(123)
rfs.feature = FSelectInstanceSingleCrit$new(
```

```
                    task = rfs.task,
                    learner = rfs.learner,
                    resampling = rsmp("holdout"),
                    measure = msr("surv.cindex"),
                    # terminator =    trm("none"),
                    terminator =    trm("evals", n_evals = 20),
                    store_models = TRUE)
fselector = fs("rfe",recursive = TRUE,feature_number = 1)
fselector$optimize(rfs.feature)
rfs.feature$result_feature_set
rfs.feature$result_x_domain
```
